# Soft monolithic infrared neural interface for simultaneous neurostimulation and electrophysiology

**DOI:** 10.1038/s41377-023-01164-9

**Published:** 2023-05-24

**Authors:** Marcello Meneghetti, Jaspreet Kaur, Kunyang Sui, Jakob F. Sørensen, Rune W. Berg, Christos Markos

**Affiliations:** 1 DTU Electro, Department of Electrical and Photonics Engineering, Technical University of Denmark, DK-2800 Kgs, Lyngby, Denmark; 2 Department of Neuroscience, University of Copenhagen, Blegdamsvej 3B, DK-2200 Kbh N, Copenhagen, Denmark; 3 NORBLIS ApS, Virumgade 35D, DK-2830 Virum, Denmark

**Keywords:** Optoelectronic devices and components, Polymers

## Abstract

Controlling neuronal activity using implantable neural interfaces constitutes an important tool to understand and develop novel strategies against brain diseases. Infrared neurostimulation is a promising alternative to optogenetics for controlling the neuronal circuitry with high spatial resolution. However, bi-directional interfaces capable of simultaneously delivering infrared light and recording electrical signals from the brain with minimal inflammation have not yet been reported. Here, we have developed a soft fibre-based device using high-performance polymers which are >100-fold softer than conventional silica glass used in standard optical fibres. The developed implant is capable of stimulating the brain activity in localized cortical domains by delivering laser pulses in the 2 μm spectral region while recording electrophysiological signals. Action and local field potentials were recorded in vivo from the motor cortex and hippocampus in acute and chronic settings, respectively. Immunohistochemical analysis of the brain tissue indicated insignificant inflammatory response to the infrared pulses while the signal-to-noise ratio of recordings still remained high. Our neural interface constitutes a step forward in expanding infrared neurostimulation as a versatile approach for fundamental research and clinically translatable therapies.

## Abstract (web summary)

A soft infrared polymer fiber (a) has been used to develop bi-directional interfaces for simultaneous infrared neural stimulation and electrophysiology (b) over several weeks (c) with minimal tissue damage (d).

## Graphical abstract

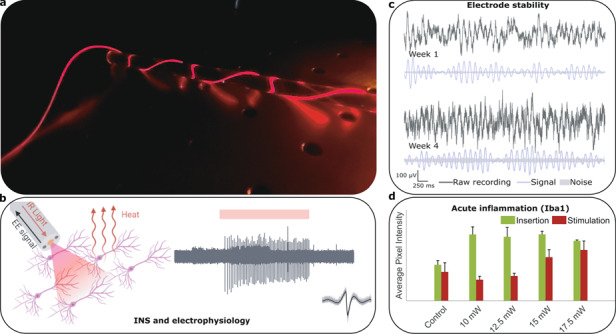

## Introduction

Understanding the biological mechanisms underlying neural circuits is one of the fundamental goals in modern neuroscience^1^. Precise and reliable tools to modulate and monitor neuronal activities are essential elements within this frame^2,3^. With the discovery of optogenetics^4,5^ and infrared neurostimulation (INS)^6,7^, light-induced control of neurons with high temporal resolution became one of the most powerful tools in neuroscience^8^. Therefore, new milestones have been set within the biotechnology community for novel devices able to deliver light in localized brain regions across the full electromagnetic spectrum^9–11^. Interrogation of the brain signals is a complex task and therefore bi-directional interfaces integrated with multiple functionalities in a single monolithic structure are of great benefit. Chronic in vivo experimentation adds an additional challenge of formation of glial scarring by the tissue’s foreign body response (FBR)^3^. Therefore, the design and development of minimally invasive devices based on biomaterials close to the Young’s modulus of the brain has attracted significant attention the past few years^2,12–14^.

While optogenetics is arguably one of the most powerful methods for high temporal resolution stimulation of neural populations, the necessity for genetic manipulation of the targeted cells adds further complexity to the process by requiring either the injection of a viral vector or the use of genetically modified animals^8,15^. INS has thus been proposed as a viable transgene-free alternative to optogenetics^7^. This method relies on the light absorption from the biological tissue at specific water-dominant regions affecting the neuronal activity through temperature-mediated processes^16^. INS offers tunable spatial resolution (by varying the operational wavelength) with the ability to affect individual axons without the need for the introduction of exogenous substances in the tissue^17,18^. While the fundamental biophysical mechanisms of INS are not yet completely understood^7,19^, it is employed successfully in several biomedical research areas, such as restoration of hearing, investigation of brain diseases, control of peripheral nerves and cardiac pacing^7^. In the past few years, the technological requirements for brain-compatible tools have been addressed for use in optogenetics to a certain extent. Several research groups have already reported innovative multifunctional brain devices such as polymer fibres and new soft planar implantable technologies^3,20–23^. However, infrared (IR) counterparts to these technologies suitable for in vivo INS in the brain have not yet been reported. IR light is normally delivered to regions deeper than the superficial cortical layers by implanting conventional silica glass optical fibres^24,25^ or silicon-based devices^26,27^. Both platforms exhibit a significant mismatch in mechanical properties with respect to the brain tissue^2,10^. Conventional step-index polymer optical fibres have been used in optogenetics and have in general found a vast amount of sensing applications in the visible regime^28,29^. However, this type of fibres suffers from significantly high absorption losses at longer wavelengths attributed to their fundamental vibrational absorption, which is inherently dependent on the chemical structure of the monomer. Consequently, the existing polymer optical fibre technology cannot be adopted for INS applications.

Here, we address these challenges of INS by developing a soft biocompatible multifunctional neural interface able to deliver IR light and record the brain activity in vivo, by thermally drawing a structured polymer optical fibre (SPOF) using non-conventional high-performance polymers. The developed SPOF devices can deliver light over a broad IR transmission spectral window spanning from 450 nm up to 2100 nm wavelength overlapping with one of the main absorption peaks of biological tissues at 1930 nm. Microelectrodes have been integrated proximal to the core of the fibre, allowing *artifact-free* electrophysiological recordings from the targeted brain region. IR pulses from a supercontinuum laser were delivered through the developed SPOF implants, activating the neuronal circuitry with minimal tissue damage. The stability performance of the integrated microelectrodes during recordings of local field potentials (LFPs) was evaluated over multiple weeks of implantation.

## Results

### Fibre design and thermal drawing process

The thermoplastic polymers suitable for thermal drawing of implantable step-index waveguides need to fulfill several optical, thermal and mechanical requirements such as refractive index (RI) difference to satisfy Snell’s law, biocompatibility, glass transition temperatures, and overlapping viscosity profiles for simultaneous thermal processing^30,31^.

The commonly used polymers in step-index optical fibres are polymethyl methacrylate (PMMA), polycarbonate (PC) and cyclic olefin copolymers (COCs) such as Topas and Zeonex^32^ (RIs and Young’s moduli listed in Table S1). Despite their relatively low optical loss in visible wavelengths (~3.5 dB/m), they have never been reported to transmit light in the 2 μm range over a few centimeters, which is a typical implantation length^14^. To fulfill the IR transmittance and flexibility requirements, we chose polysulfone (PSU) and fluorinated ethylene propylene (FEP) copolymer as materials for the fibre core and cladding, respectively. PSU is a sulfur-based polymer that provides good transmission in the IR compared to its more conventional optical polymer counterparts^14^. This high-performance polymer is characterized by high hydrolytic resistance and has been demonstrated to be highly biocompatible^33^. FEP, on the other side, is one of the softest biocompatible thermoplastics with Young’s moduli as low as 0.48 GPa, making it a promising material for suppressing the FBR while being optically transparent^34,35^. In addition, the large RI contrast between the two polymers leads to a very large numerical aperture (NA) (Fig. S1a for the measured RIs), which is known to have an important role during neurostimulation^36^. The PSU and FEP viscosity-temperature profiles were characterized using standard rheometry and found to be thermally compatible and thus suitable for fibre co-drawing (Fig. S1b).

**Fig. 1:**
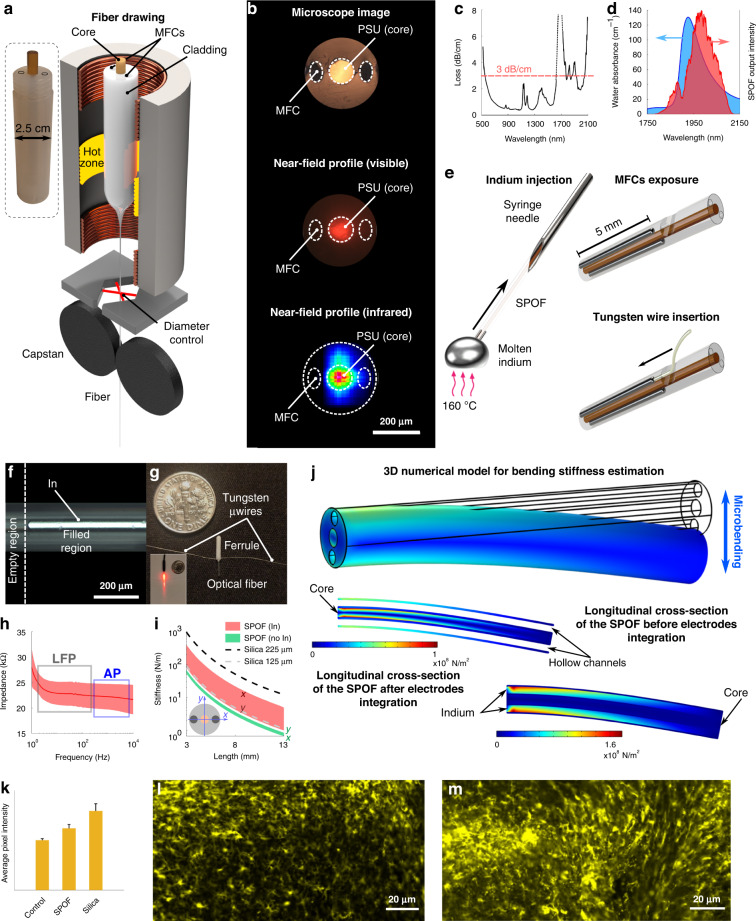
Fabrication and characterization of the IR multifunctional neural interfaces. **a** Schematic of the drawing process for the polymer optical fibre used to develop of the neural interfaces. In the inset, a picture of the preform used for the drawing, with the hollow channels on the sides of the preform highlighted by black ellipses. **b** From top to bottom: microscope image of the structure of the fabricated optical fibre and near-field profiles of the output light at both visible and IR wavelengths. **c** Attenuation spectrum of the SPOF in the spectral range spanning from 500 to 2100 nm wavelengths. The shaded regions show the spectral ranges with losses < 3 dB/cm. **d** Overlap between the absorbance of water (extracted from ref. ^58^) and the spectrum transmitted from the SPOF. The same light source and optical setup were used in the INS experiments shown later in the article. **e** Schematics of the fabrication steps to integrate electrodes in the optical fibre and connectorize them for EE recording. **f** Microscope image of the interface between the indium-filled and the hollow part of a microfluidic channel exposed for connectorization. **g** Assembled multifunctional probe after connectorization. Inset: visible light transmitted through the connectorized probe. **h** Impedance spectrum and corresponding standard deviation (shaded) of the integrated electrodes in the frequency ranges of LFPs and APs. **i** Simulation of the bending stiffness of the SPOF, both with and without microfluidic channels filled with indium and compared with the most commonly used silica optical fibres in neuroscience. **j** 3D numerical model of the distribution of von Mises stress in the SPOF during bending (top), showing the maximum concentration of stress shifting from the core (center) to the side channels (bottom) due to the injection of indium. **k** Average fluorescence intensity of the IBA 1 marker in 350 × 142 µm ROIs along the implantation region of SPOF-based interfaces and 225 µm silica fibres, compared with control animals (N slices > 7, 4 weeks implantation). **l**, **m** Representative images of IBA 1 marked microglia recruited at the implantation site of the SPOF interfaces and silica fibres, respectively

The macroscopic template of the fibre, known as preform, was prepared by machining commercially available bulk polymer rods. Two side channels next to the core of the fibre were introduced for the subsequent integration of the electrodes. The preform was then heated and scaled down into a fibre using an in-house draw tower facility (Fig. 1a, Supplementary Video S1). During the fibre fabrication, the drawing parameters of feeding/pulling speed were adjusted to obtain a ~60 times diameter reduction, corresponding to a core size of ~105 µm, in order for the produced interfaces to be compatible with standard patch cables and components such as cannulae and adaptors typically used in animal experiments. Since the diameter of the preform was 25 mm, this draw-down rate resulted in a fibre external diameter of 420 µm (Fig. S2).

A single preform was enough to produce several hundred metres of SPOF with uniform core/cladding ratio along the entire length (Supplementary Video S1). The observed standard deviation of the fibre diameter was found to be ~1% (Fig. S2). Minor deformations in the microfluidic channels (MFCs) can be attributed to the capillary forces acting on the FEP cladding during drawing combined with the blade fingerprint introduced by the cleaving (top image, Fig. 1b). The near-field profiles of the propagation modes in the visible (central image Fig. 1b) and IR (2 μm) band (bottom image, Fig. 1b) demonstrate the strong confinement of the light in the core, which is crucial for enhanced localization during INS. The optical attenuation spectrum of the fibre was measured by cut-back technique after coupling in it the light from a coherent broadband supercontinuum source. The observed losses were lower than 3 dB/m in a broad band from 550 to 1650 nm and in several bands in the 1750–2100 nm region. Similar values of losses have been previously reported to be compatible with the use of fibre-based devices for neural applications^14^. By filtering the source with a long-pass filter (LPF) with a cut-off at 1800 nm, the output spectrum from the SPOF overlaps with the main absorbance peak of water at ~1930 nm, as shown in Fig. 1d, which is the INS band used in this work. The losses of the fibre in this spectral band have a minimum of 2 dB/cm.

### Preparation of the multifunctional neural interfaces

Electrophysiology is a critical modality to directly record the neuronal response under different neurostimulation methods. Low-impedance electrodes were integrated in the SPOF, to enable simultaneous INS at the 2 μm spectral region and high-quality extracellular electrophysiology (EE) recordings. Short SPOF segments corresponding to the implantation length were connected to a syringe needle, which was used to create negative pressure in the channels to infiltrate molten indium (left, Fig. 1e). This specific metal was selected for the integrated electrodes due to its relatively low melting point (~155 °C) and low Young’s modulus compared to other metals (Table S2). Indium has been recently reported to be a suitable electrode material for in vivo EE recording over several weeks^21^. The choice of high-performance polymers with high melting points (>200 °C) was critical in achieving the desired outcome, since it allowed the use of any metal with a melting point lower than the materials of the fibre as electrodes. Therefore, the SPOF could sustain the contact with the molten metal without any damage. The hollow channels were uniformly filled with indium, without introducing any structural deformation during the infiltration (Fig. 1f). The fibres were then extracted from the syringe and placed under an optical microscope to mechanically expose the hollow channels from the side (Fig. 1e, top right) and allow the insertion of 50 µm thick tungsten wires (Fig. 1e, bottom right). Once the tungsten wires were in contact with the indium electrodes, they were fixed in position with cyanoacrylate adhesive. The SPOF neural interfaces were finally packaged for the in vivo experiments in ceramic ferrules (1.25 mm diameter, 6.4 mm length) (Fig. 1g) for back-end connectorization. The impedance of the electrodes was characterized in the 1–10,000 Hz range using electrochemical impedance spectroscopy in a three-terminal configuration (*N* = 6). Values <35 kΩ were recorded in both the local field (<250 Hz^37^,) and the action potential (AP) region (300–7000 Hz^38^), as shown in Fig. 1h. The individual measurements of impedance and phase are presented in Fig. S3. These values are well below the 1 MΩ at 1 kHz maximum value recommended for EE^10^.

The bending stiffness of the device is directly linked with the tissue damage upon implantation and fixation to the skull^14^. In order to evaluate the stiffness characteristics of the proposed implants, we employed a finite element method (FEM) model for different lengths based on the cross-sectional geometry. Our calculations were performed both for the as-drawn SPOF and for the implants with integrated metallic electrodes, by changing the mechanical parameters of the hollow channels between air and indium. Despite the presence of metallic electrodes and the large overall diameter of the fibre, the calculated stiffness was found to be comparable to or even lower than the one of the silica fibres with diameters most commonly used in optogenetics applications (125 and 225 µm external diameter) (Fig. 1i). Since, unlike conventional glass fibres, the SPOF and implants are not centrosymmetric their stiffness is represented as a shaded area to account for its angular dependence. The lower edge of the area represents the minimum stiffness level, resulting from a bending force parallel to the axis connecting the center of the electrodes in the fibre’s cross section. In contrast, the upper edge represents the maximum, i.e., a bending force perpendicular to that same axis. The large spread between the two directions in the model with indium-infiltrated channels is due to the fact that almost all of the internal von Mises stress of the fibre during bending is concentrated in the metal inclusions (the non-centrosymmetric part of the structure), while in the model with hollow channels the stress is concentrated in the centrosymmetric core (Fig. 1j). To further validate the enhanced biocompatibility of the SPOF-based interfaces with respect to standard optical fibres, we performed an immunohistochemistry (IHC) evaluation of the recruitment of microglia at the implantation. Animals implanted with our interface (*N* = 2) and standard 225 µm silica fibres (*N* = 2) were transcardially perfused, and their brains out-dissected, after 4 weeks of implantation. The brains were then sliced and stained for activated macrophage marker ionized calcium-binding adaptor molecule 1 (Iba-1) detection. Both pristine animals and surgery-free hemispheres from implanted animals were used as control samples (*N* = 5). The fluorescence intensity in regions of interest (ROIs) along the longitudinal fibre insertion was used as an indicator of the gliosis induced in the brain by the implants (Fig. 1k). Measurements from several slices suggest a difference in average intensity between the three groups of samples, with the SPOF interfaces performing statistically better than silica fibres (*p* ~ 0.1, Fig. S4). The results are in good accordance with the literature predicting lower foreign body response with the use of soft materials in brain interfaces^3,14^. Representative images of Iba-1 fluorescence in brains implanted with the two different kinds of fibre are presented in Fig. 1l, m.

### In vivo simultaneous INS and electrophysiology

**Fig. 2:**
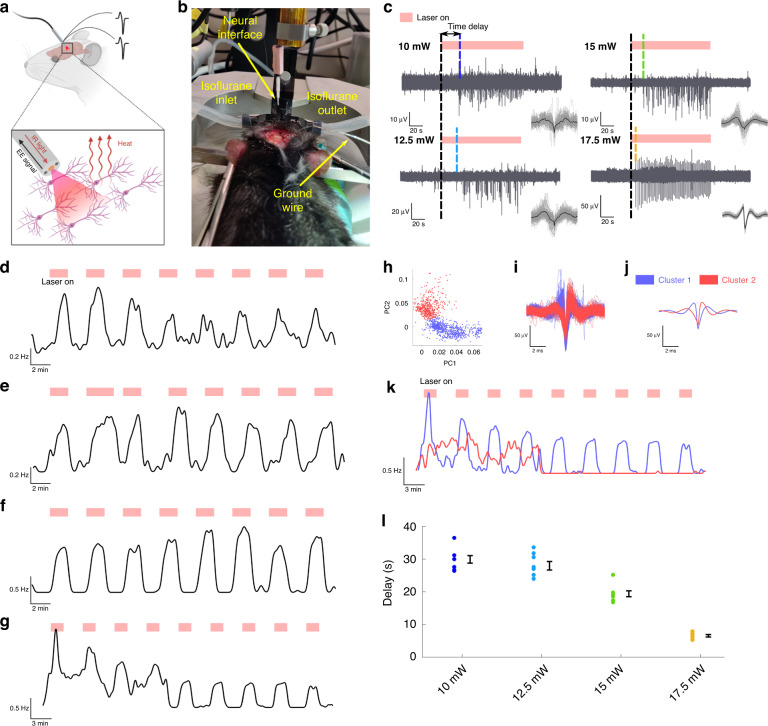
Opto-electrophysiological interrogation of the brain. **a** Concept of the experiment: delivering IR light with the developed interface to generate heat-induced variations in neural activity while conducting simultaneous electrophysiology based on the integrated electrodes. **b** Picture of the surgery for the insertion of the implant in the brain, taken immediately prior to the insertion. **c** Neural response to two minutes stimulation cycles for laser powers ranging from 10 to 17.5 mW; the dashed lines indicate the time delay between the beginning of the stimulation and the onset of the neural activity; the overlap of the recorded spikes (grey) and their average (black) shown as insets are also reported, with the respective scale bars, in Fig. S8. **d**–**g** Average firing rates over several stimulation cycles, calculated by convoluting the spikes temporal positions extracted by the signal in Fig. S6 with a Gaussian kernel, for stimulation powers of (**d**) 10 mW, (**e**) 12.5 mW, (**f**) 15 mW, and (**g**) 17.5 mW. **h** Gaussian mixture model clustering of the two first principal components for the spikes recorded over several stimulation cycles at a power of 17.5 mW. **i**, **j** Overlap and average shape, respectively, of the all the spikes recorded at 17.5 mW after clustering. **k** Average firing rates, calculated by convoluting the spikes’ temporal position with a Gaussian kernel, of the two units individuated by principal component analysis. **l** Delays between the beginning of the stimulation and the beginning of the recording activity for all stimulation cycles at different powers. The average and standard error are presented on the side of each series

Water absorption is the main factor introducing a thermal response to IR light in biological tissues. Our neural interfaces were tested in vivo to evaluate their performance for stimulation of the neural activity in the cortex of two adult rats with IR light overlapping with one of the strongest water absorbance peaks in the 2 μm region (Fig. 1d). During stimulation, the integrated electrodes recorded the EE response of neurons to the IR light (Fig. 2a) delivered >1 mm deep into the brain following implantation of the interfaces in anesthetized animals during a terminal procedure (Fig. 2b). The INS protocol consisted of 2 min illumination periods, similar to ref. ^26^, interspersed by 2–3 min recovery periods. This investigation was repeated at four distinct cortical locations, as shown in Fig. S4, using four different average power levels (10, 12.5, 15 and 17.5 mW). Since the brain tissues had to be further analyzed by IHC to evaluate the laser-induced damage (as presented in the following section), we avoided any positional micro-adjustments once the implant was in the desired region. This way, the surgery-related damage that might have masked the laser-induced one was minimized. The developed multifunctional neural interface was verified by recording EE signals at the aforementioned four different INS powers to investigate the neuronal dynamics in response to light-induced heating (Fig. 2c). The spike activity was recorded consistently and repeatably for each power level over several stimulation cycles (Fig. S6). The temporal locations of the recorded spikes have been convoluted with a Gaussian kernel having a width (6σ) of 10 seconds to calculate the average firing rate (Fig. 2d–g)^39^. While for all the INS powers the average firing rate consistently increases during stimulation at each cycle with respect to the endogenous activity, for the maximum power (17.5 mW) we observe relatively high values also in the resting periods for the first 20 min of recording. In order to further investigate this increased neuronal activity, principal component analysis has been performed on the recorded spikes. Gaussian mixture model clustering of the first two principal components allowed us to identify two separate unites with distinct spike shapes (Fig. 2h–j). By recalculating the average firing rate of the individual units, we can attribute the activity inconsistency at 17.5 mW with respect to the other powers to the presence of one unit (Cluster 2) exhibiting a mostly endogenous behavior that was unaffected by the stimulation (Fig. 2k). At the highest power of 17.5 mW, we recorded the potentials and clearly identified single-unit neuronal activity without need for further processing. In contrast, during the recording at lower powers, the presence of recording artifacts in form of a low frequency tail after each spike, attributed to a saturation of the analog filters, required further digital processing of the data (high pass filter, 300 Hz) to reconstruct the shape (Fig. S7). For each of these power, the high pass filter was applied to the sets of spikes appearing in the stimulation periods shown in Fig. 2c (Fig. S8) and to the complete sets of spikes from the full recordings (Fig. S9). Moreover, to reduce the noise introduced by the low frequency tails, a digital low-pass frequency filter was applied to improve the visualization of the recordings for the three lower laser powers in Fig. 2c. While effectively suppressing noise, this data post-processing introduced a reduction in the plotted intensity and a slight change in the spikes’ shape, though leaving the temporal position of the spikes unaltered (Supplementary Text 1, Figs. S7 and S10). Furthermore, the presence of a stimulation-activity time delay and the shape of the signal suggest that the recorded signal originated from the IR-modulated neural activity and not from any side laser, electrical or thermal artifacts. This is also validated by the fact that, when contact between the electrodes and the stimulated neurons could not be established, no stimulated activity was recorded despite all other conditions such as recording setup, depth of insertion and optical power being the same (Fig. S11).

While INS-driven neural activation is often reported in the literature to be in the millisecond timescale^18,40^, we instead observed a seconds-long time delay between the light delivery and onset of the stimulated activity. This can be explained by the power dependent time profile of temperature variations in tissue requiring several seconds to reach a plateau corresponding to a few °C temperature increase at the recording location^26^, as visualized in Fig. S12. This fact can be further exacerbated by the short pulse lengths (picoseconds) used for the stimulation, which are known to induce both thermal and stress confinement (Supplementary text 2) that might delay the temperature increase. At relatively low optical powers, i.e., relatively low temperature variations, the stimulation threshold could be close to the plateau value, causing the observed delay. The observed power dependence of the response time, which decreases from ~30 to 7 s as the output power increases from 10 to 17.5 mW (Fig. 2l), is also in agreement with this hypothesis. This effect was verified by repeatedly measuring the brain activity over several stimulation cycles, and confirming that the delays observed at 17.5 and 15 mW are significantly different (*p* < 0.05) from the ones observed at lower powers (Fig. S13).

Since INS offers high spatial resolution for targeted stimulation, it was recently employed to map mesoscale brain connectomes using functional magnetic resonance imaging (fMRI) in anesthetized animal (cat)^19^. To investigate the time-resolved dynamics of the brain during the previously described seconds-long response time of the electrical activity, we also conducted fMRI during INS at ~15 mW. The experiment was conducted in the motor cortex of an anesthetized rat to visualize the brain region. The requirement of relatively short acquisition times (~2 s) to temporally resolve the onset dynamics of the stimulation, limited the maximum achievable spatial resolution (0.5 × 0.5 × 1 mm) of the obtained images. However, it was possible to verify the presence of blood-oxygen-level-dependent (BOLD) signal fluctuations temporally correlated with the illumination in the voxels close to the fibre tip (Supplement text 3 and Fig. S14).

### Evaluation of laser-induced damage

**Fig. 3:**
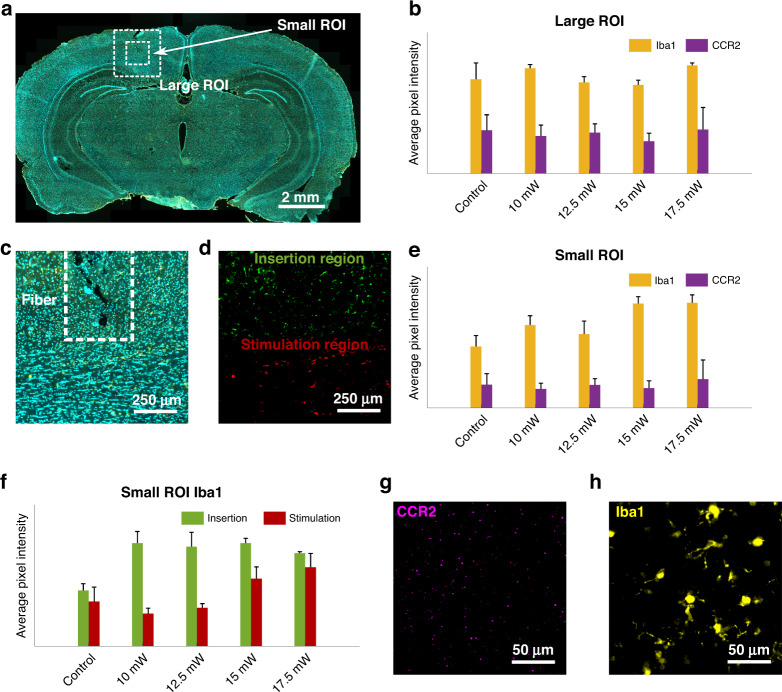
Infrared laser damage evaluation. **a** Microscope image of a brain slice used for IHC, stained with Iba-1 (yellow) and DAPI (cyan), with the ROIs used for analysis indicated as dashed lines. **b** Average fluorescence intensities for Iba-1 and CCR2 in the large ROI (*N* slices > 5). **c** Magnification of 2a showing the small ROI only; the dashed lines indicate the position of the fibre during stimulation. **d** Colorized depiction of a small ROI’s subdivision into insertion and stimulation regions (Iba-1 channel only). **e** Average fluorescence intensities for Iba-1 and CCR2 in the small ROI (N slices > 5). **f** Average fluorescence intensities for Iba-1 in the insertion and stimulation regions of the small ROI (N slices > 5). **g**, **h** Magnified images showing microglia and monocytes in the implantation region, respectively

The INS experiments performed in this investigation use a new protocol with a supercontinuum laser (broadband, picosecond long pulses in the IR) delivered in the brain tissue, as opposed to the monochromatic lasers normally used in literature^18,19,26^. It is thus important thus to investigate the neuronal damage after INS. This assessment was conducted by post-experimental transcardial perfusion of the rats (*N* = 2), out-dissection of the central nervous system, slicing of the brain and IHC analysis. Due to the terminal nature of the experiment, the brain slices were stained with two widely used markers to identify acute inflammation: Iba-1 and monocyte marker C-C chemokine receptor 2 (CCR2). Pristine rat brains (*N* = 2) were similarly perfused, dissected and analyzed as a control. Multiple brain slices were analyzed from each animal. We subsequently used the fluorescence intensity in square regions surrounding the SPOF tip as an indicator to quantify the tissue damage. To visualize the effects of the procedure as a whole, in terms of both the insertion and the laser-induced thermal load, the average pixel intensity was firstly measured in a region of interest centered on the fibre’s tip and comprising the whole length of the implant (large ROI) (Fig. 3a). However, a large portion of unaffected tissue was included in the analysis, leading to the lack of any statistically significant difference between the control brains and the brains that underwent surgery and INS (Fig. 3b). We therefore reduced the region of interest to 1 × 1 mm, while keeping it centered on the fibre’s tip (small ROI, Fig. 3a, c) and, since the focus of this work is on the laser-induced damage, this small ROI was split perpendicularly to the fibre into an insertion region and a stimulation region (Fig. 3d), to separate the effects of the laser heating from the mechanical damage caused by the implantation surgery. In the small ROI, while the intensity related to CCR2 remained similar for all the sample groups, an increase in Iba1-related intensity was identified for the stimulated samples, showing the presence of acute inflammation induced by the procedure (Fig. 3e). In the sub-regions of the ROI, as shown in Fig. 3f, statistically significant increase in Iba-1 intensity with respect to control (*p* < 0.05, Fig. S15) was observed in the insertion region for all the stimulated samples while no significant difference was observed between the different powers, compatibly with the fact that the insertion procedure was identical in all brain regions. On the other hand, a power-dependent increase of the intensity was measured in the stimulation region, with the value for the two higher powers being significantly larger than the one for the two lower ones (*p* < 0.05, Fig. S16), where the IR thermal load is expected to be directly proportional to the laser power (Fig. 3f). However, none of the stimulated region exhibit values significantly larger than control, showing that any induced inflammation is close to negligible (Fig. S16). The fluorescence signals from the Iba-1 and CCR2 markers are shown in Fig. 3g and h, respectively.

### Measurement of local field potentials using the integrated electrodes

**Fig. 4:**
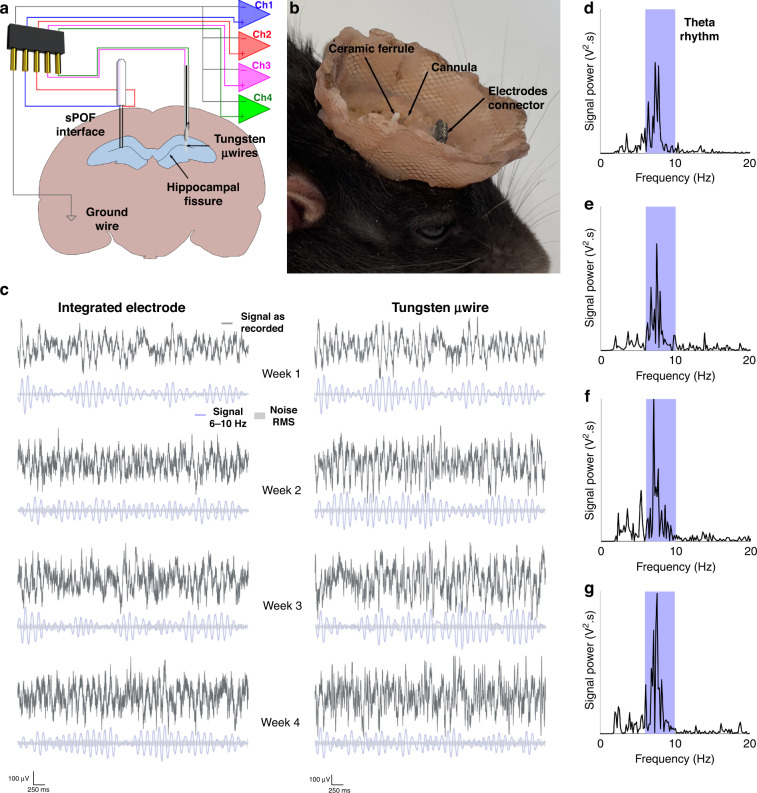
Chronic local field potential measurement in the hippocampus. **a** Schematic representation of the implants and recording setup. **b** Chronic implant of a multifunctional neural interface and a steel cannula with tungsten µwires in the brain. **c** Dark grey: 5-second-long EE recordings of LFPs in the hippocampus from an integrated indium electrode and a tungsten µwire over four weeks. Light blue: 6–10 Hz (Theta rhythm) component of the recorded signal. Light grey shading: root mean square amplitude of noise (defined here as recorded signal outside the 6–10 Hz range). **d**–**g** Power spectra of the indium electrode recordings shown in Fig. 4c for weeks 1, 2, 3 and 4 respectively, with the 6–10 Hz region corresponding to theta rhythm highlighted in light blue

Given that the main aim of the proposed interfaces is to enable chronic INS studies, we investigated the long-term stability of the interface between the integrated electrodes and the brain. Since LFPs have attracted a strong interest in the last decade from the community for adaptive neuromodulation^41,42^, as a verification method we quantified the LFPs’ SNR over four weeks of recording in behaving animals (Supplementary Video S2). Hence, the neural interfaces were chronically implanted in the left hippocampus of two rats. Tungsten microwires (µwires), which are highly stable electrodes and commonly used in the literature for LFP recording^43–45^, were also implanted in the right hippocampus as benchmark (Fig. 4a, b). The flexible µwires (50 µm diameter) were placed in stainless steel cannulae similar in size to our interfaces, to ensure correct positioning during implantation (Fig. 4b). Representative 5 s periods with no motion-induced artifacts were extracted from each recording, using a high-pass filter (1 Hz) to remove baseline drifts and DC offsets (Fig. 4c), full 10 min recordings for week 4 in Fig. S17). As shown in Fig. 4d–g, the recorded oscillations had the main components of their power spectrum in the 6–10 Hz region, directly related to the emergence of hippocampal theta rhythm correlated to the animal’s movements^46,47^. In Fig. 4c, the signal components falling in the theta rhythm frequency range are also presented below each recording, as well as the root mean square amplitude of noise (defined as all signal falling out of the 6–10 Hz range). The trend of the SNRs calculated from the 5-s periods over time show a similar performance between the integrated electrodes and the tungsten µwires in terms of stability (Fig. S18). The SNR was defined as the ratio between the integrated signal powers of theta rhythm and noise over the 5-second periods, normalized by the bandwidth in Hz of the frequency windows. The brains were sliced and imaged at the end of the four weeks to evaluate the gliosis induced by the SPOF fibre implant. Fig. S19 visualizes the difference, regarding the scarring left in the brain, between the two implants used for this recording, which have considerably different shape and mechanical properties.

## Discussion

We have developed an IR neural interface for simultaneous INS and electrophysiology based on soft materials. The initial fibre templates were fabricated by high-performance thermoplastics using a thermal drawing process. This method ensures the scalability, as well as the future possibility to easily reduce the device’s diameter to further suppress inflammatory response, since the bending stiffness can be reduced by reducing the cross-sectional area of the fibre (Fig. S20). Electrodes that were monolithically integrated in the fibre had a low measured impedance, leaving space for further reduction of their surface without compromising the recording performance. Furthermore, the high thermal performance and hydrolytic stability of the polymers used to fabricate the presented SPOF make the device suitable for sterilization both by steam- and dry-heat^48,49^, which are between the most cost-effective and diffused sterilization methods for medical devices^50^, contributing thus to the efforts for sustainable biotechnology.

Our neural interfaces were verified by in vivo simultaneous INS and electrical recording in anesthetized and behaving animals. A supercontinuum laser covering a broad transmission spectrum enabled stimulation at the 2 µm region water absorption peak. Our protocol can efficiently and reproducibly evoke neural activity at different powers, with minimal power-dependent inflammation in the brain, confirming the existing literature on INS being a safe approach for neurostimulation under controlled conditions^7,17^. The integrated electrodes allowed us to obtain EE recordings with low noise (<30 µV peak-to-peak). The use of IR light efficiently removed the artifacts commonly arising from the Becquerel effect when using shorter (visible) wavelengths^51^.

The hollow channels in the SPOF were functionalized with metal electrodes to develop a protocol for the straightforward integration and back-end connectorization of the electrical recording functionality in our neural interfaces. It should be noted that the resulting indium-tungsten electrodes are highly reproducible as shown by the impedance spectra. Furthermore, the infiltration approach of metals in microstructured fibres has the potential for scalability to larger numbers of electrodes as it has been shown already for the development of tunable devices based on hybrid photonic crystal fibres^52^. Freely behaving animal experiments showed that both the electrode connections and the brain-electrode interfaces are as efficient and reliable as the tungsten electrodes widely used in the field of neuroscience.

In conclusion, infrared neurostimulation is a powerful tool to investigate the function of small structures in the brain by affecting both excitatory and inhibitory circuits^19^. Our soft neural interfaces could greatly advance the use of this technique in deep brain regions and chronic settings, to gain a better understanding of neural mechanisms at a circuit level. Furthermore, due to the biocompatibility and sterilizability of the used materials, they may also increase the potential for translation of the findings towards the development of new therapies for neural diseases in humans.

## Materials and methods

### Mechanical and optical characterization of the materials

The viscosity-temperature profiles of PSU (core) and FEP (cladding) were measured in the 40–300 °C temperature range using a rotational rheometer (TA, Discovery Hybrid HR-2). Thin rectangular samples of dimensions 2 mm thick, 10 mm wide and 25 mm in length were loaded into the clamps of the system and locked with screws. A constant upward force is applied in the axial direction during heating to analyze the solid samples. We chose an axial force of 1 N with a sensitivity of 0.1 N. The refractive indices of the materials were measured in bulk hot-embossed samples in the 210–1000 nm range using an ellipsometer (J.A.Woollam, VASE) with a 5-nm resolution^53^.

### Thermal drawing of the IR fibres

Commercially available rods of FEP and PSU were purchased by Goodfellow, UK and annealed in a drying oven for more than two weeks at 120 °C. The preform was assembled by machining the rods to obtain a 15 cm long FEP cylinder with a total diameter of 26.2 mm, a 7 mm central hole and two 6 mm side holes, and a solid PSU cylinder with a diameter of 7 mm. The fibre was thermally drawn using a three-zone furnace by stabilizing the temperature of the central one at 260 °C. The preform was heated and pulled at a speed of 2 m/min The fibre diameter was controlled during the process using a high-precision laser detector (<0.1 µm resolution).

### Integration of the indium electrodes

The integration of the indium electrodes in the SPOF started by cutting 5-cm-long pieces of fibre and partially inserting them in 18 G syringe needles. The fibre pieces were subsequently sealed in position using epoxy resin (G14250, Thorlabs), which was left to dry overnight. Afterwards, a short length of indium wire (diameter 1.5 mm, 99.999% purity, Goodfellow, UK) was melted on a heating plate and infiltrated into the side channels over >5 mm by using a syringe. The fibre pieces were then separated from the needles with a blade, and the filling quality was verified with an optical microscope, under which the non-filled length of the hollow channels was exposed on both sides by using a scalpel. Tungsten wires (diameter 50 µm, purity 99.999%, Goodfellow UK) were manually inserted until contact with the indium was achieved and were finally glued in position with small drops of cyanoacrylate adhesive (Loctite).

### Fabrication of the tungsten µwire cannulae

As a first step to fabricate the tungsten µwire cannulae used as a benchmark for LFPs recording, the coating was removed from a 300 µm length at one end of 10-cm-long pieces of insulated tungsten wires (A-M Systems, 50 µm bare diameter, 100 µm coated diameter). The wires were then inserted in a 26 G syringe needle acting as a guide for the implantation. The wires were finally fixed with glue with a ~500 µm offset between the wire tips, to avoid short-circuiting during the implantation.

### Microscope imaging of the fibre and near field profiles

To image the cross section of the fabricated fibres, we first cleaved short lengths of the fibres and ground them on different grits of polishing paper, starting from 30 μm down to 0.3 μm. The polished fibre cross-sections were then imaged using a Zeiss Axioscan A1 microscope, equipped with an Axiocam 305 colour camera, in transmission mode. The image of the fibre’s side in Fig. 2g was acquired using the same microscope in reflection mode.

The near field profile of the propagating light in the SPOF was recorded in the visible by coupling light from a Thorlabs M660L4 red LED and imaging the collimated output light with an IDS U3-3680XLE camera. A similar procedure was used for the near IR near field profile, using a BKtel 1.55 µm custom nanosecond laser as a light source and recording the output with a Thorlabs BP109-IR2 slit scanning beam profiler.

### Broadband attenuation measurements

The SPOF’s optical transmission was measured by butt-coupling the output light from a broadband supercontinuum source (NKT SuperK Extreme) in the SPOF’s core, using a silica patch cable with the same core diameter of 105 µm (Thorlabs M61L01). An identical patch cable was used to collect the light at the fibre’s output end and measure its spectrum with a SP320 scanning spectrometer from Instrument Systems. The measurement was repeated after cutting the fibre at different lengths while keeping the input end fixed, and results at each wavelength were fitted to an exponential curve to obtain the optical losses in dB/cm.

### Numerical modelling of bending stiffness

To evaluate the flexibility of the fabricated SPOF, a FEM analysis model based on COMSOL Multiphysics was used to simulate the bending and calculate the corresponding bending stiffness. 3D FEM models of the fibre, both with and without the indium electrodes, were developed starting from the cross-sectional geometry of the drawn fibre. For the modeling of standard commercial fibres, uniform silica cylindrical shapes were used as geometry, since there is almost no variation in mechanical properties between core and cladding. Similarly to the one used in ref. ^36^, the numerical model is based on fixing one end of the fibre while applying a force perpendicular to the fibre’s axis to the other end. The simulated diameters were 400 µm for the SPOF and 125 and 250 µm for the silica optical fibres.

### Impedance spectroscopy

The impedance characterization of the integrated electrodes was conducted in phosphate-buffered saline (PBS) using a Hioki IM 3590 chemical impedance analyzer in a three-terminal configuration (Fig. S21). A tungsten microwire wrapped around a glass rod acted as a counter electrode and an Ag/AgCl electrode was used as a reference electrode. The frequency range used for the measurements was 1–10,000 Hz.

### Pre-implantation procedures

Wild-type adult Long Evans rats (Charles River Laboratories) were used to perform the experiments. All surgical procedures were aseptic. Anesthesia was induced in the rats using isoflurane gas (1–3% in oxygen), followed by head shaving and skin disinfection with chlorohexidine followed by 70% ethanol. The animals were then moved to a stereotaxic frame with a heating pad and oximeter to maintain and monitor the animal. After confirming surgical anesthesia by loss of toe pinch response, the head was fixed using ear bars. Lidocaine was administered subcutaneously at the incision location, and ocryl gel was applied to maintain eye moisture. A sterile drape was placed to cover the entire body, followed by further cleaning of the head with 70% ethanol. Finally, an incision was made to expose the skull. Anesthesia was maintained throughout the surgery by constant administration of isoflurane gas at the abovementioned concentration.

### Acute implantations

For acute implantations (*n* = 2), a surgical drill was used to expose four distinct regions of the brain cortex (locations shown in Fig. S5) and a posterior brain region for grounding the animal with a silver wire. A minimum of 4 mm distance between the individual regions was maintained. The multifunctional neural interface was inserted sequentially in the different regions at a depth of >1 mm, using a single smooth motion to avoid additional damage to the tissue, to perform the simultaneous INS and EE experiments at different powers. At the end of the experiments, the rats were deeply anesthetized by an intraperitoneal injection of pentobarbital, followed by transcardial perfusion^54,55^ with a solution of 4% paraformaldehyde (PFA) in PBS.

### Chronic implantations

For chronic implantations, 3% hydrogen peroxide was applied to the skull, clearing connective tissue, followed by thorough washing with saline solution. Electrocautery was used to stop any bleeding. A 3D printed baseplate was adhered to the skull using Metabond (Parkell) cement, and a fine sheet of copper mesh was glued onto the baseplate. Using the stereotaxic robot and drill, a small hippocampal area (2 mm diameter) was exposed on each hemisphere (anterioposterior 3.6 mm, mediolateral 2 mm, dorsoventral 3 mm from dura mater). A multifunctional neural interface was implanted in the left hemisphere while a cannula with tungsten µwires was implanted in the right hemisphere of the animals used for LFP electrophysiology. Silica fibres with a diameter of 225 µm were implanted in animals used for chronic gliosis evaluation. The skull was grounded using a silver wire in contact with the cerebrospinal fluid. All the regions were sealed using silicon adhesive (Kwik-Sil, World precision instruments) followed by cyanoacrylate adhesive and dental cement. Finally, the copper mesh was molded into a crown, covered by dental cement to smoothen the surface (Fig. 3a). The crown served two functions: the protection implants from damage during the movement of the rat and the minimization of electromagnetic noise while recording electrical signal from the brain (by acting as a Faraday cage). After the surgery, the rats were orally treated with buprenorphine (0.2 mg mixed with 1 g Nutella) every 12 h for three days. Furthermore, Carprofen (5 mg/kg), and Baytril (5 mg/kg) were administered subcutaneously every 24 h for 5 and 10 days, respectively. The conditions of the animals were monitored 2–3 times per day over the first 3 days and once daily for the following week.

### In vivo IR neurostimulation

Light from a broadband supercontinuum source (NKT SuperK Extreme, 1–20 MHz repetition rate, average power up to 6 W) was filtered using a long-pass filter (cut-off wavelength of 1800 nm) and coupled to a silica fibre patch cable. The patch cable was then connected to a multifunctional neural device using a ceramic mating sleeve. The power at the output of the devices was recorded prior to each insertion using a Thorlabs S401C thermal power sensor. Following insertion, light was delivered continuously for 2 min intervals, followed by 2–3 min resting periods. Four distinct regions of the brain cortex were stimulated at different optical powers (10, 12.5, 15 and 17.5 mW) for each animal, starting with the highest power and proceeding sequentially towards the lowest one.

### Electrophysiological recordings

The acute EE recordings during INS were conducted by connecting the exposed tungsten wires from the neural devices to the headstages of differential amplifiers (DP-311A, Warner Instruments). The signal was recorded through an ADInstruments PowerLab 4/26 DAQ using LabChart 8 software. For all recordings, the internal high-pass and low-pass filters of the amplifiers were set at 300 Hz and 10 kHz respectively, and the sampling rate was set at 20 kHz.

The chronic electrophysiological recordings of LFPs in freely behaving animals were performed using an Intan RHD 2000 USB interface board equipped with a RHD 2164 amplifier, at a sampling rate of 20 kHz. The data was recorded using Intan’s RHX Data Acquisition Software.

### Immunohistochemistry

After both acute and chronic experiments, the animals’ brains were removed and kept in PFA for 4 h before being transferred to sucrose 30% (w/v) for cryoprotection. A cryostat was used to slice coronal brain sections (25 μm thickness) that were immediately collected on superfrost plus glass slides (Thermo Fisher Scientific GmbH, Germany) for IHC following a procedure similar to^56,57^. The slices were washed in PBS and incubated a room temperature (2 h) with a blocking solution (5% bovine serum albumin, 0.3% Triton X-100, 5% fetal bovine serum, 1% PBS). Anti-CCR2 (1:100, rabbit polyclonal, Thermo Fisher Scientific PA5-23037) and anti-Iba-1 (1:1000, rabbit polyclonal, Wako 019-19741) primary antibodies were applied to the slices, followed by overnight incubation at 4 ^o^C. Afterwards, the slices were rewashed in PBS and secondary antibodies (donkey anti-rabbit, Alexa Fluor 633, 1:500, Invitrogen) were applied, together with 4’,6-Diamidino-2-Phenylindole, Dihydrochloride (DAPI, Thermo Fisher Scientific, D1306) was applied at a concentration of 1:1000. Finally, after 2 h at room temperature, the glass slides were mounted using DAKO mounting medium and imaged using a Zeiss Axioscan Z1 microscope (×20 magnification).

For the IHC analysis of acute experiments, images were initially analyzed using the Fiji open-source image processing package, based on ImageJ2. All images were opened at full resolution in the software, and the marker and DAPI channels were separated. The marker channel images were then cropped to two different square ROIs (referred to as “large” and “small” in the text) of 2 × 2 mm and 1 × 1 mm sizes, respectively, centered around the tip of the inserted SPOF. After discarding the images found to be out of focus or presenting too large artifacts, a threshold was applied to eliminate the signal from out-of-plane cells, and the maximum pixel value was adjusted to increase contrast. The threshold and maximum were kept as constants for all images obtained from slices stained with the same marker, while variating between different markers. The average pixel intensity was then calculated using Fiji. For a more precise analysis, the small ROIs for slices stained for the Iba-1 marker were further divided into 0.5 × 1 mm ROIs (insertion and stimulation), splitting them across a line normal to the fibre insertion direction.

IHC analysis of chronic experiments was performed using a similar methodology with Zeiss ZEN software, using 314 × 142 µm rectangular ROIs along the length of the implantation region.

### Data processing and representation

The data processing, analysis and plotting were performed in the Matlab 2019b suite.

## Supplementary information

Revised supporting information

Supplementary Video S1

Supplementary Video S2

## Data Availability

The data that support the findings of this study are available from the corresponding author upon reasonable request.
