# Supplementary material for: Soft monolithic infrared neural interface for simultaneous neurostimulation and electrophysiology
Source: Light Sci Appl. 2023 May 24;12:127. doi: 10.1038/s41377-023-01164-9 (PMC10209158; doi:10.1038/s41377-023-01164-9)
Supplement: Supplementary file 1 — Revised supporting information [file 41377_2023_1164_MOESM1_ESM.docx]

# Supplementary Information for:

**Soft monolithic infrared neural interface for simultaneous neurostimulation and electrophysiology**

Marcello Meneghetti^a,b,^*, Jaspreet Kaur^b,^ ^†^, Kunyang Sui^a,b,^ ^†^, Jakob F. Sørensen^b^, Rune W. Berg^b^, Christos Markos^a,c,^*

*Corresponding authors. Email: [mamen@dtu.dk](mailto:mamen@dtu.dk) (M. Meneghetti), [chmar@dtu.dk](mailto:chmar@dtu.dk) (C. Markos)

^†^ These authors contributed equally

^a^ DTU Electro, Department of Electrical and Photonics Engineering, Technical University of Denmark, DK-2800 Kgs. Lyngby, Denmark

^b^ Department of Neuroscience, University of Copenhagen, Blegdamsvej 3B, DK-2200 Kbh N, Denmark

^c^ NORBLIS ApS, Virumgade 35D, DK-2830 Virum, Denmark

| **Material** | **Young’s modulus (GPa)** | **Refractive index (@ 635 nm)** | **References** |
| --- | --- | --- | --- |
| PSU | 2.48 | 1.63 | [33] |
| FEP | 0.48 | 1.34 | [36] |
| PMMA | 3 | 1.49 | [36,59] |
| PC | 2.4-2.6 | 1.58 | [59,60] |
| COC | 1.3-3 | 1.51-1.54 | [59,60] |
| Silica | 72.3 | 1.46 | [61] |
| Silicon | 165 | 3.87 | [62] |

### Table S1: Optical polymers.

Optical polymers have typically low Young’s moduli when compared with broadly used optical materials such as silica and silicon, and exhibit a broad range of refractive indices. In this table, these physical properties are compared between the optical materials used for the SPOF (in red) and other commonly used materials in multifunctional neural interfaces.


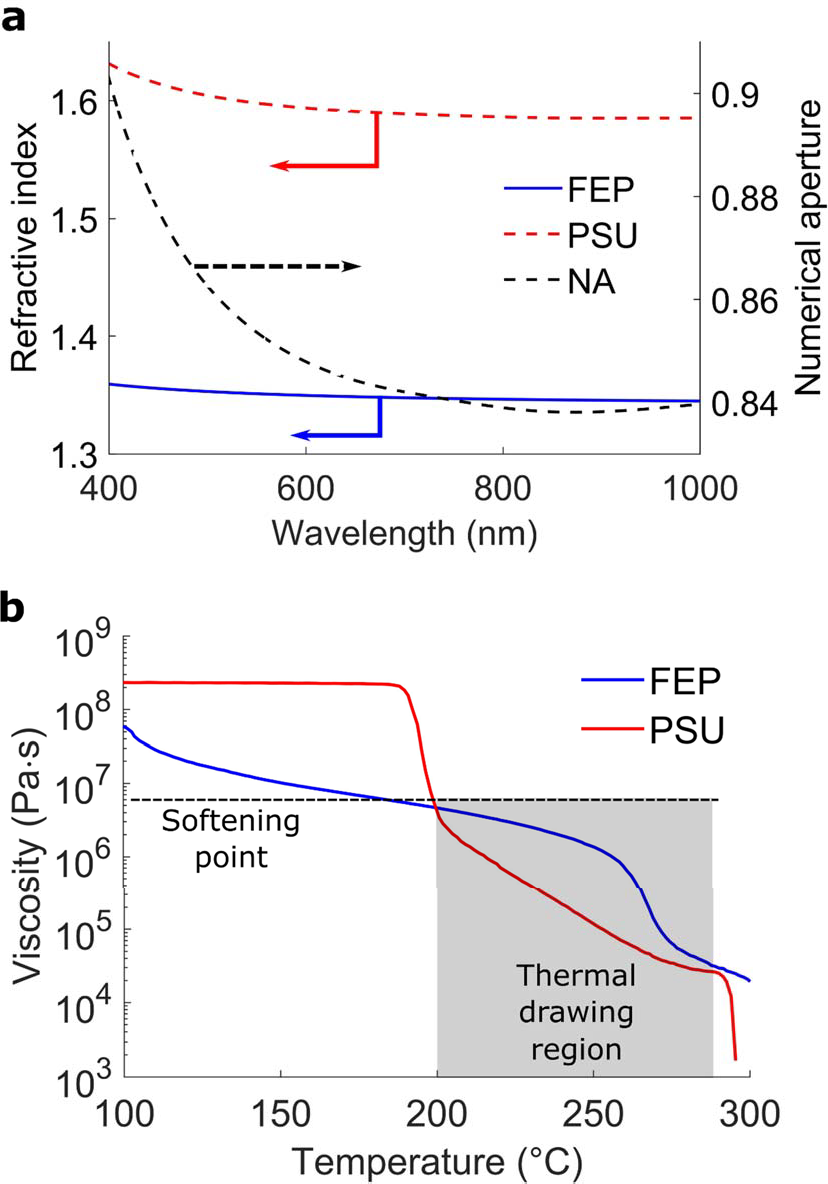


### Fig. S1: Optical and thermal characterization of the polymers constituting the sPOF.

The materials chosen to fabricate the SPOF have a large difference in refractive index, leading to a large NA, and are thermally compatible for fibre drawing. The measured refractive indexes (solid lines) calculated NA are presented in (**a)**, while the viscosity profile of the two polymers are presented in (**b**) The dashed line in (**b**) indicates the Littleton Softening Point (10^6.6^ Pa·s).

### Supplementary Video S1.

Spooling of the uniform POF on a drum in our in-house draw tower facility.


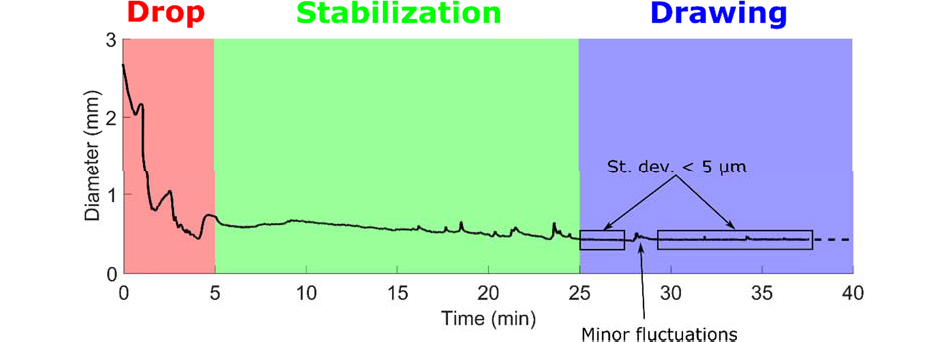


### Fig. S2: Temporal evolution of the fibre diameter during thermal drawing.

After the falling of the drop and the adjustment of the drawing parameters (feeding speed, drawing speed and temperature), tension and diameter stability have been reached at a drawing speed of ~5 m/min. Aside from minor fluctuations, the diameter after stabilization has been constant (~420 µm) with a standard deviation of ~1.1%.

| **Material** | **Young’s modulus (GPa)** | **References** |
| --- | --- | --- |
| Indium | 12.7 | [21] |
| Tin | 44.3 | [63] |
| Tungsten | 345 | [63] |

### Table S2: Electrode materials.

Indium, the electrode material used for the neural interface described in this paper, is softer than other commonly used metals. This table compares its Young’s modulus (red) with the ones of tin and tungsten.


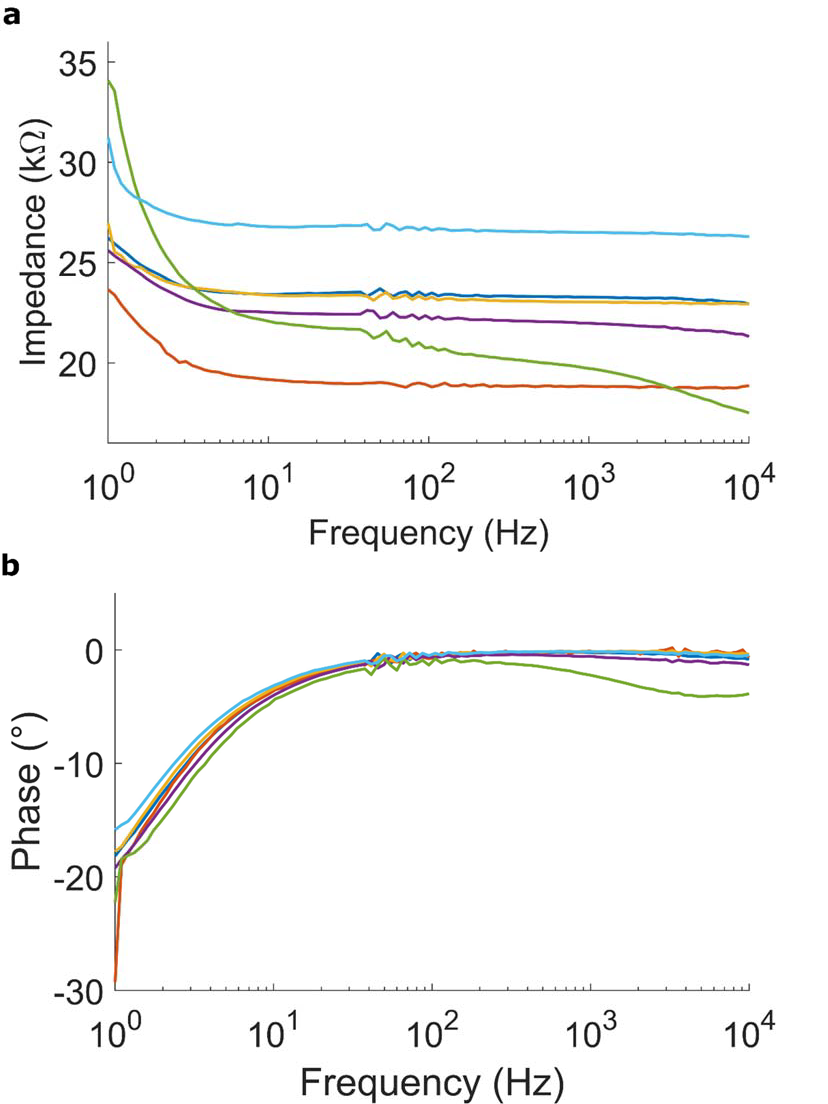


### Fig. S3: Impedance spectroscopy of the indium-tungsten electrodes.

The measured impedance of the electrodes integrated in the neural interface is well below the limit for detection (1 MΩ at 1 kHz), as shown in Fig. 1h in the main manuscript. Here, we present 6 individual measurements of impedance (**a**) and phase (**b**).


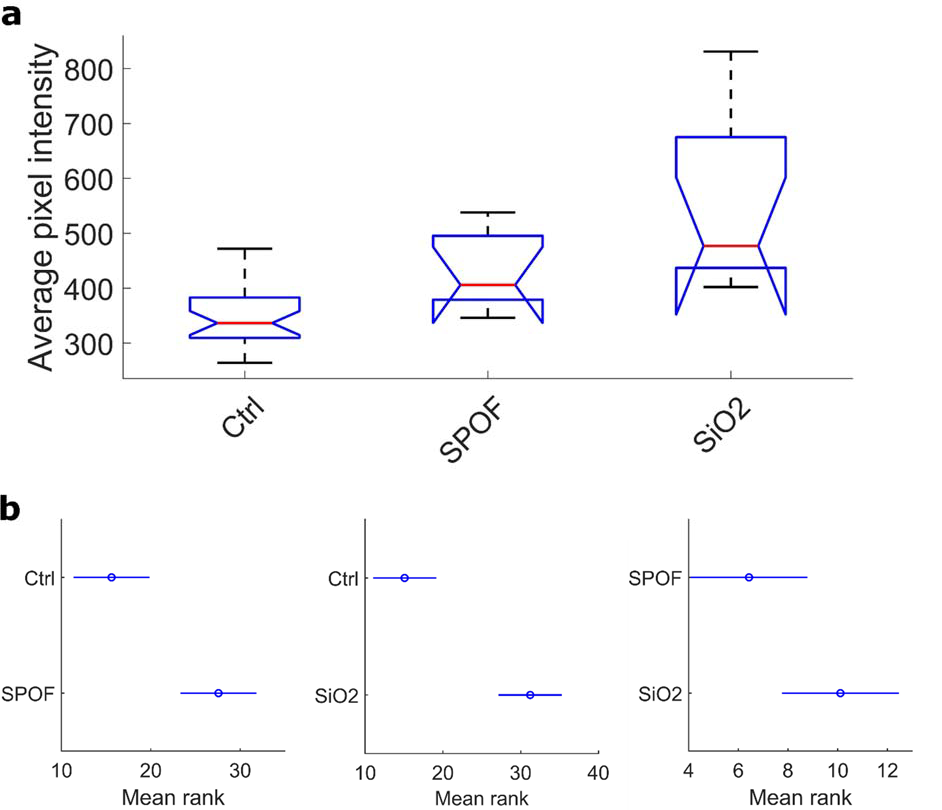


### Fig. S4: Statistical significance of the immunohystochemical analysis in the insertion region.

The average IBA1 intensity in the implantation region is significantly (p<0.05) higher than control in all sample populations from animals that underwent chronic implantation. This has been verified by a Kruskal-Wallis test **(a)**, and pairwise Tukey honest significance tests have been performed for protection against type I errors **(b)**. Due to the low number of samples, the null hypothesis could not be rejected at p<0.05 by the tests for the SPOF-silica pair. However, the relatively low p- value (~0.1) and the strong accordance of the result with existing literature still suggest a lower amount of recruited microglia in the SPOF-implanted population with respect to the silica-implanted one.


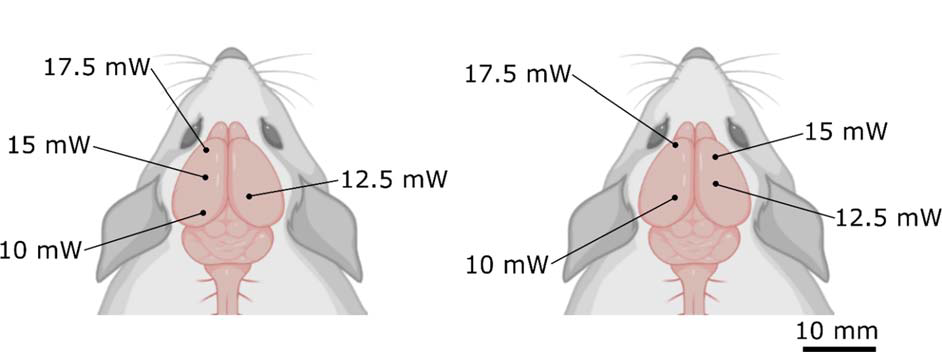


### Fig. S5: Cortical regions used for acute INS experiments.

To be able to individually evaluate the laser damage at different powers, the acute stimulation and electrophysiology experiments reported in this work have been performed at separate cortical locations. Here, we present those locations for the two animals considered (insertion depth of 1 mm).


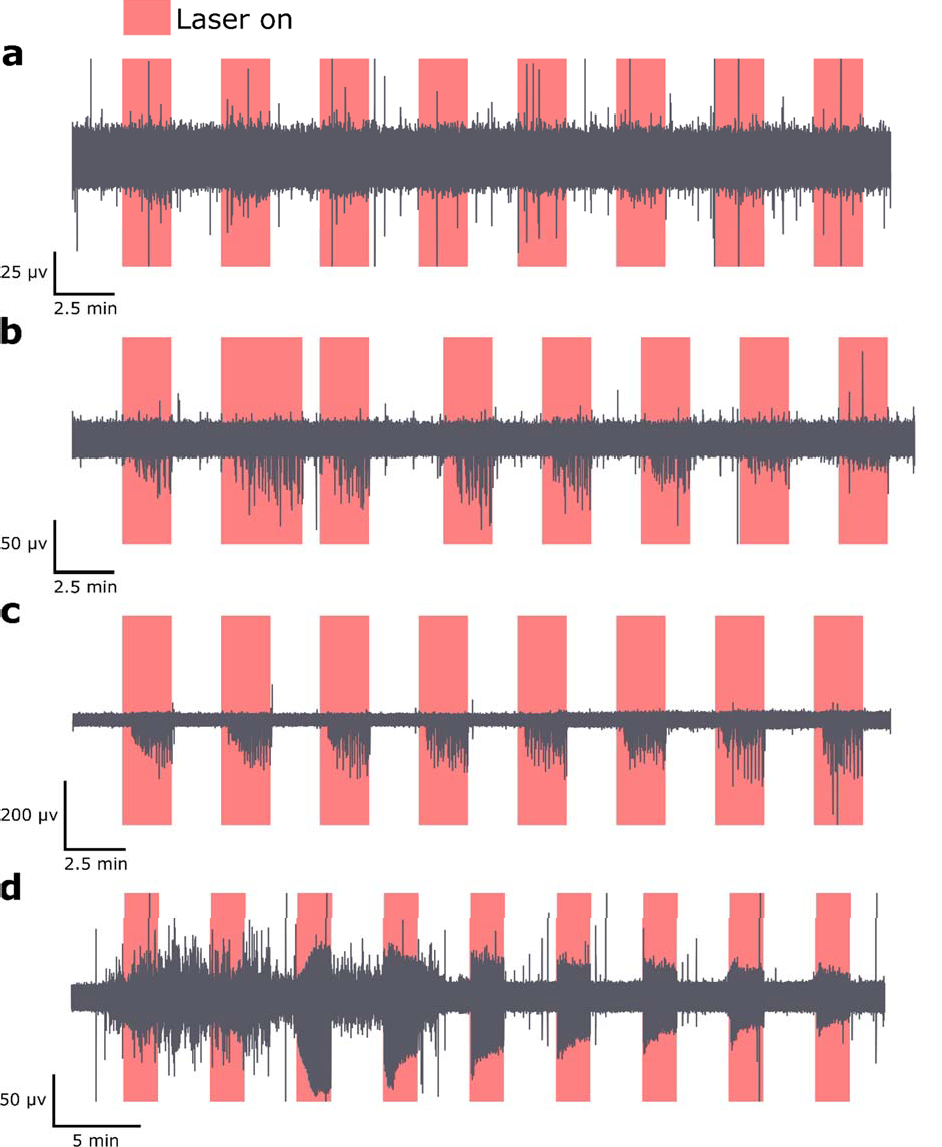


### Fig. S6: Full INS-electrophysiology recordings.

For all acute experiments, several stimulation periods of the duration of two minutes each were used to ensure the repeatability of the results. Here, we present the full recordings for stimulation powers of **a)** 10 mW, **b)** 12.5 mW, **c)** 15 mW,

**d)** 17.5 mW, whitout any digital post-processing.


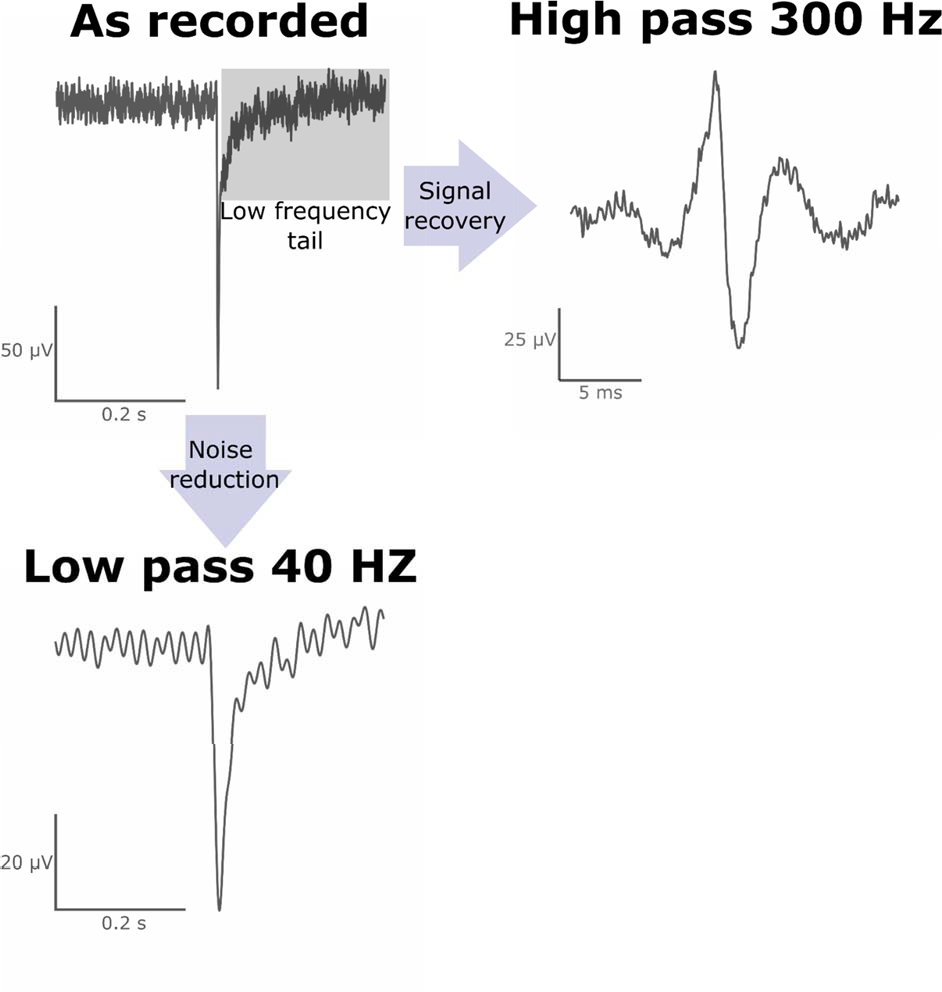


### Fig. S7: Effect of digital filters on spikes with low-frequency tail.

The low frequency tail of the spikes observed in some of the recordings requires a further digital elaboration of the signal. Low pass filtering can be used to strongly reduce the background noise intensity and improve the visualization, while high pass filtering allows to recover the shape of the individual spikes.


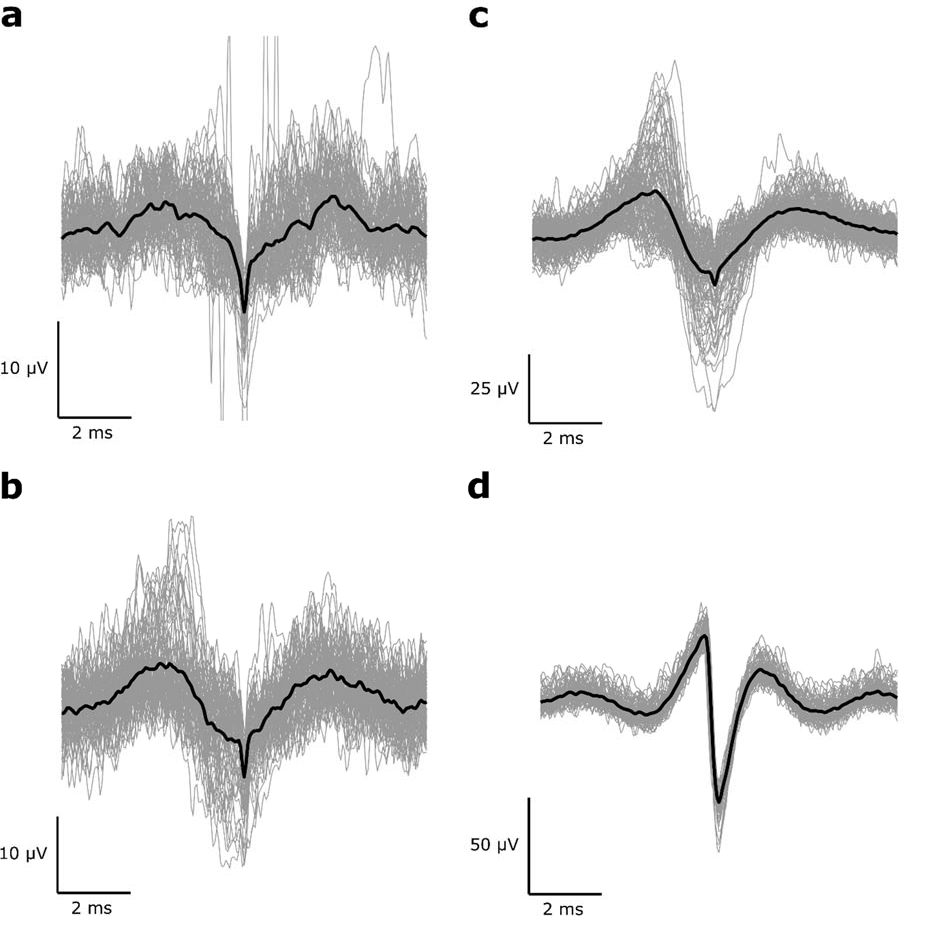


### Fig. S8: Individual spikes recorded in a single stimulation cycle.

In the stimulation periods presented in Fig. 2c it was possible to distinguish individual neural spikes. Here we present the overlap of these spikes (grey) and their average shape (black) for stimulation powers of **a)** 10 mW, **b)** 12.5 mW, **c)** 15 mW,

**d)** 17.5 mW. A 300 Hz high pass filter was applied in **a**,**b** and **c**.


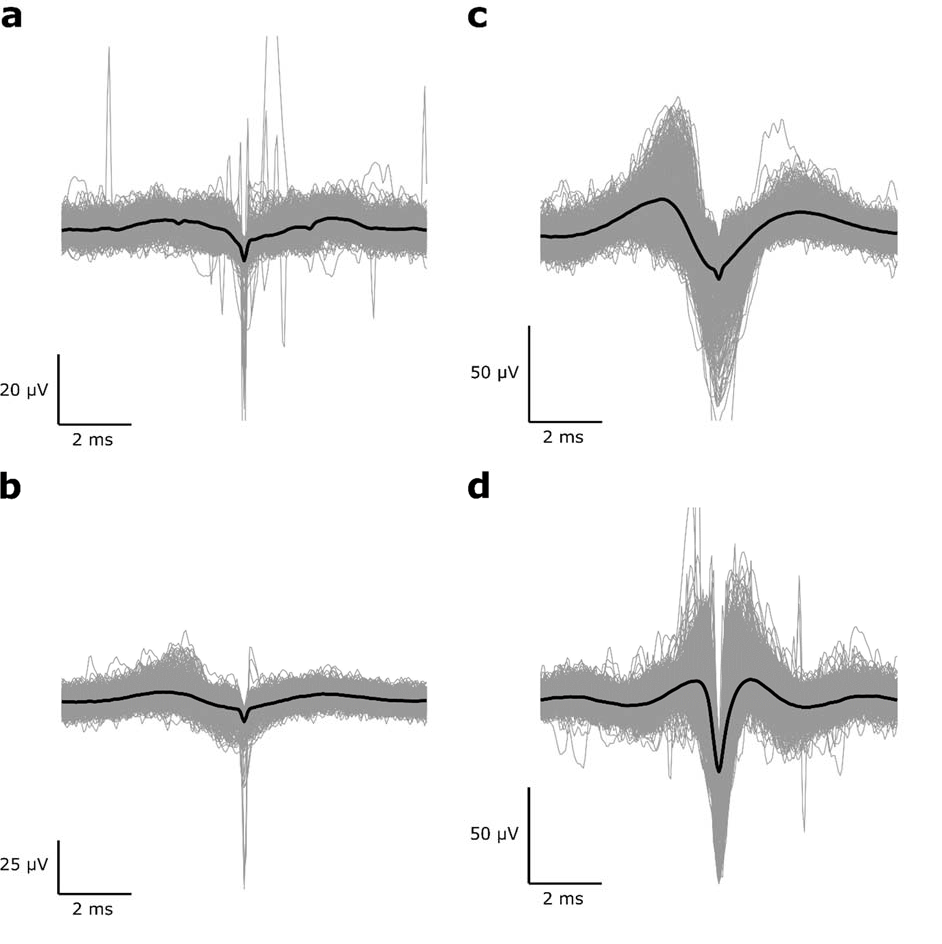


### Fig. S9: Individual spikes recorded over several stimulation cycles.

From the full recordings presented in Fig. S5 it was possible to extract a large number of individual spikes. Here we present the overlap of these spikes (grey) and their average shape (black) for stimulation powers of **a)** 10 mW, **b)** 12.5 mW, **c)** 15 mW, **d)** 17.5 mW. A 300 Hz high pass filter was applied in **a**,**b** and **c**.

## Supplementary Text 1: Post-processing of electrophysiological recordings

A notch filter (50 Hz) has been digitally applied to all electrophysiological recordings presented in the main text. Additionally, to highlight the neural activity for the stimulations performed with 10, 12.5 and 15 mW optical power presented in Fig. 2, a low-pass digital filter with a cut-off frequency of 40 Hz has been applied prior to plotting (Fig S7). The lower signal-to-noise ratio in these recordings has been attributed to a combination of suboptimal contact between the electrodes and the neurons and the saturation of the analog filters integrated in the recording setup used, which also caused the low frequency tail of the spikes visible in Fig.S7. The full recordings after the application of this filter are shown in Fig. S10. As visible in figures S7-S9, The application of a 300 Hz digital high-pass filter allowed to recover the information on the neural signal. For the digital processing, all the filters were second order Butterworth filters.


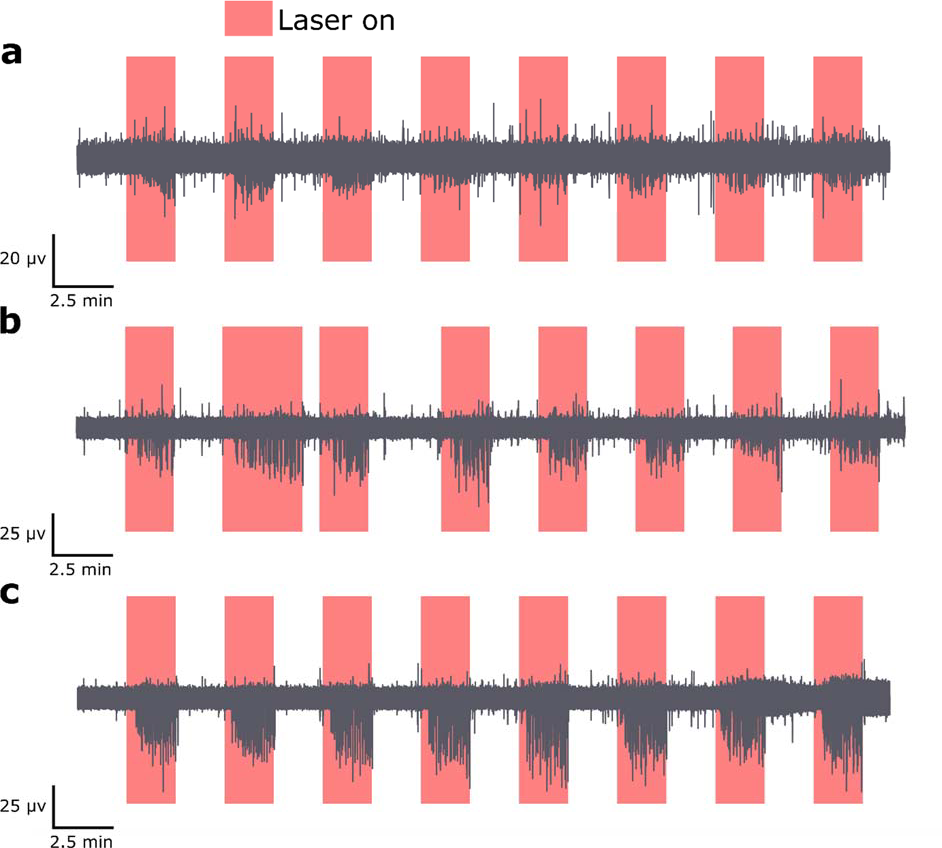


### Fig. S10: Effect of low-pass filtering for highlighting the neural signal.

For the recordings presented in Fig. S5a-c, a low-pass filter allowed to reduce the impact of the low frequency tails shown in Fig. S5 and improve the visualization of the spikes, especially for lower powers. Those recordings have been replotted in this figure after the application of a 40 Hz digital filter.


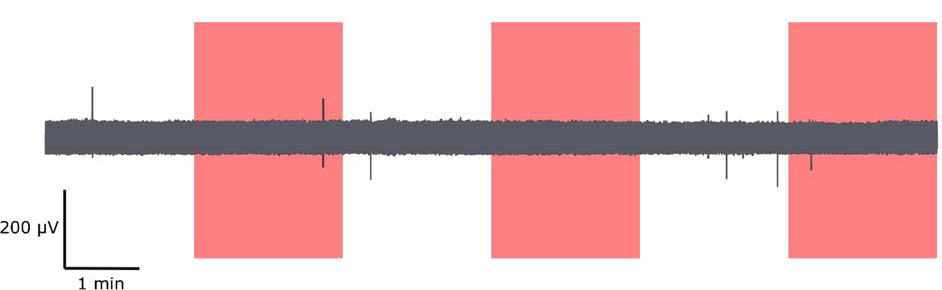


### Fig. S11: Stimulation experiment with no established electrical contact.

Due to the acute nature of the experiments performed on simultaneous INS and electrophysiology, and to the fact that to minimize insertion damage to the brain no microadjustments to the position of the SPOF-based neural interface were performed, in some cases it has been impossible to achieve electrical contact between the electrodes and the neurons. The absence of stimulated signal in these cases confirms that the signal recorded when contact was achieved is neural, rather than related to thermal or electrical artifacts. Here we show three stimulation cycles in which, due to the absence of contact, no stimulated activity was recorded when illuminating the neurons with an optical power of 17.5 mW.


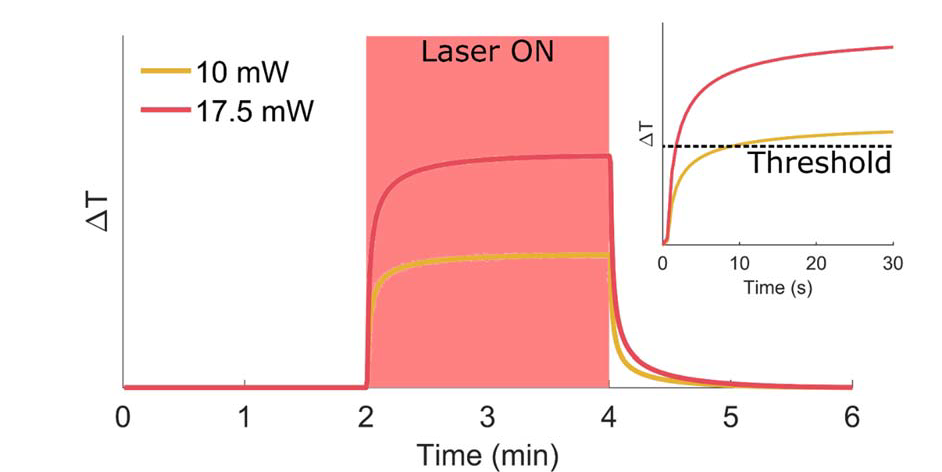


### Fig. S12: Representation of power-dependence of time delay.

During interaction with infrared light, the temperature of brain tissue increases rapidly in the first few seconds and gradually reaches a plateau (stabilization) afterwards. Since the level of the plateau is proportional to the laser power, once a temperature threshold for neural activation is fixed, it is possible to visualize the power-dependence of the time delay between light delivery and stimulated neural activity. Here, we show the typical time dependence profile of temperature increase in neural tissues during INS (in the simplified case of single wavelength, continuous wave laser) for two different powers. In the inset, zoom on the temporal region following the onset of the stimulation.

## Supplementary Text 2: Thermal and stress confinement conditions

Photomechanical effects in laser-tissue interaction are enhanced when thermal and stress confinement conditions are met, i.e. when the laser pulse width *t_p_* is smaller than the thermal and acoustic relaxation times *t_th_* and *t_ac_* of the tissue. These relaxation times can be calculated as [64]:

and

*d2 t_th_* = *4X*

*d t_ac_* = *cs*

Where *d* is the smallest dimension of the irradiated volume, *χ* is the tissue thermal diffusivity coefficient (0.129 mm^2^/s in brain tissue [65]) and *c_s_* is the speed of sound (1550 m/s in brain tissue [66]). For the experiments described in this manuscript, where the light delivered to the brain has a relatively broad spectrum (1800-2100 nm), *d* cannot be directly defined. For wavelengths that are less absorbed from the brain (i.e. absorption coefficient *α* < 95 cm^-1^ for water [67]), *d* is equal to the fibre core diameter (i.e. 105 μm). For wavelengths with *α* > 95 cm^-^ 1, which constitute ~30% of the delivered light, *d* is instead equal to the penetration depth of light in the tissue (i.e. the distance at which the intensity of light drops to 1/e). As the maximum value of *α* for water in the 1800-2100 nm band is equal to 130.6 cm^-1^ [58], it is however possible to determine that *d* is strictly comprised between 76 and 105 µm. The corresponding ranges for the relaxation times are ~10-100 ms for *t_th_* and ~45-70 ns for *t_ac_*. The supercontinuum laser, with *t_p_* being picosecond range, therefore meets the conditions for both confinement regimes (thermal and stress).


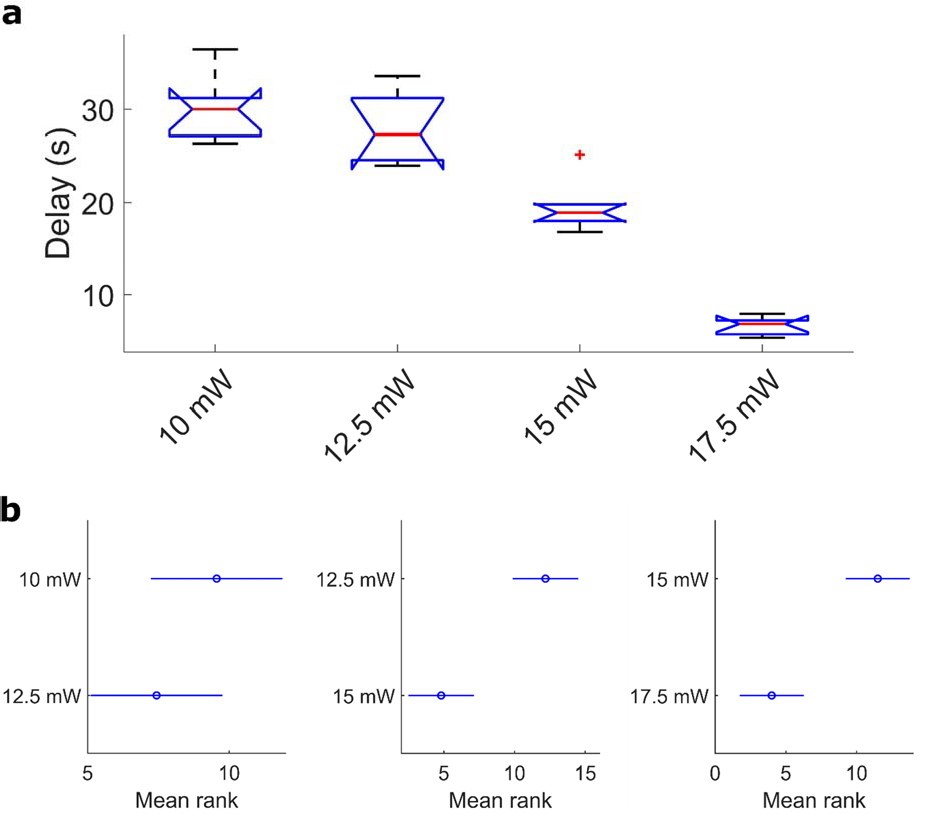


### Fig. S13: Statistical significance of the difference between time delays.

The power dependence of time delay on optical power presented in Fig. 2l is statistically significant (p<0.05) for powers higher than 12.5 mW. This has been verified by a Kruskal-Wallis test **(a)**, and pairwise Tukey honest significance tests have been performed for protection against type I errors regarding the difference between low and high power **(b)**.

## Supplementary Text 3: fMRI imaging and analysis

The fMRI data was acquired on a 9.4 T scanner (Bruker Biospec 9.4/30 USR AVANCEIII/MRI) during a stimulation protocol similar to the one described for simultaneous INS and electrophysiology (15 mW, 2 minutes OFF – 2 minutes ON cycles, 5 repetitions).

Structural images were obtained using a TurboRARE sequence with an in-plane resolution of 0.137x0.137 mm (5 slices, 0.5 mm thickness), a repetition time of 3500 ms and an echo time of 34 ms. The bandwidth for these measurements was 50 kHz, and the matrix size was 256x256 (field of view 3.5x3.5 mm). Functional images were obtained with a spin echo EPI sequence at a voxel size of 0.5x0.5 (in plane) x 1 (slice thickness) mm (3 slices) in a 64x64 matrix. The echo and repetition time were of 35 ms and 2000 ms respectively, the bandwidth was 250 kHz, and the field of view was of 32x32 mm.Functional MRI images were opened as stacks in the Fiji software, and the slices centered around the fibre insertion region were isolated. The temporal profiles of voxels in a 5x7 voxel region of interest close to the fibre tip were then exported from Fiji as numerical vectors for further analysis.


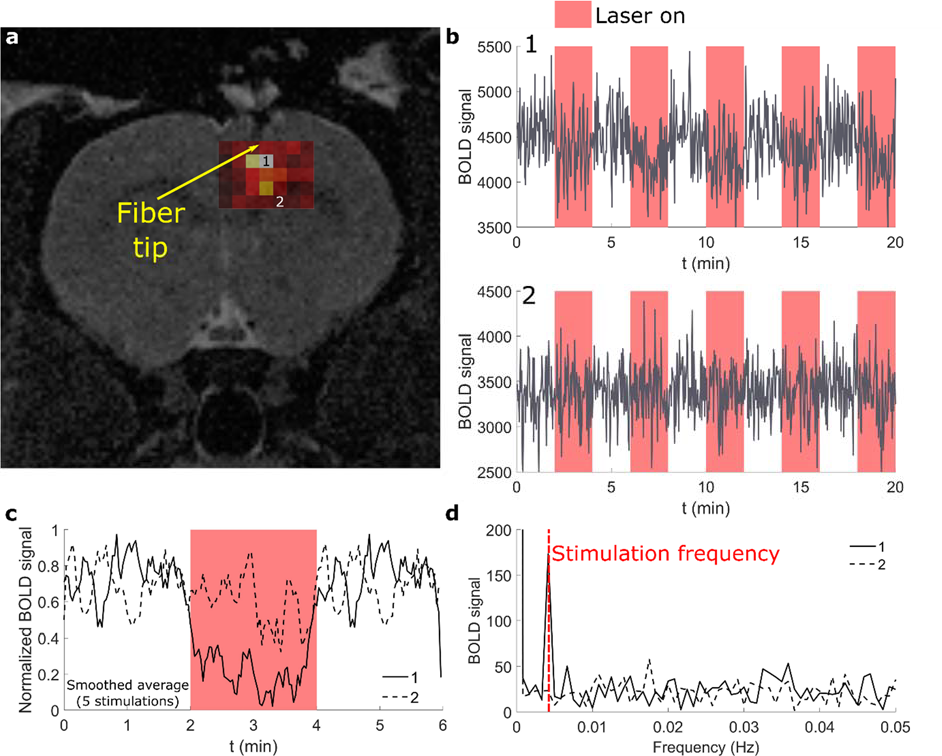


### Fig. S14: Visualization of INS by functional magnetic resonance imaging.

To visualize the effects of INS at the location of the fibre tip, we employed fMRI scanning to analyze the oscillations in BOLD signal in the targeted region. For some of the voxels, it was possible to verify a strong temporal correlation between these oscillations and the infrared stimulus, as presented in this figure. (**a**) Overlap between a structural MRI image and the signal intensity at the stimulation frequency obtained by fast Fourier transform of the time profile of an EPI scan. (**b**) Temporal profile of the BOLD signal during the scan for voxels 1 and 2 are indicated in (**a**). (**c**) Comparison of the smoothed average of BOLD signal over 5 stimulations between voxel 1 and voxel 2. (**d)** Comparison of the spectral components of BOLD signal between voxel 1 and voxel 2.


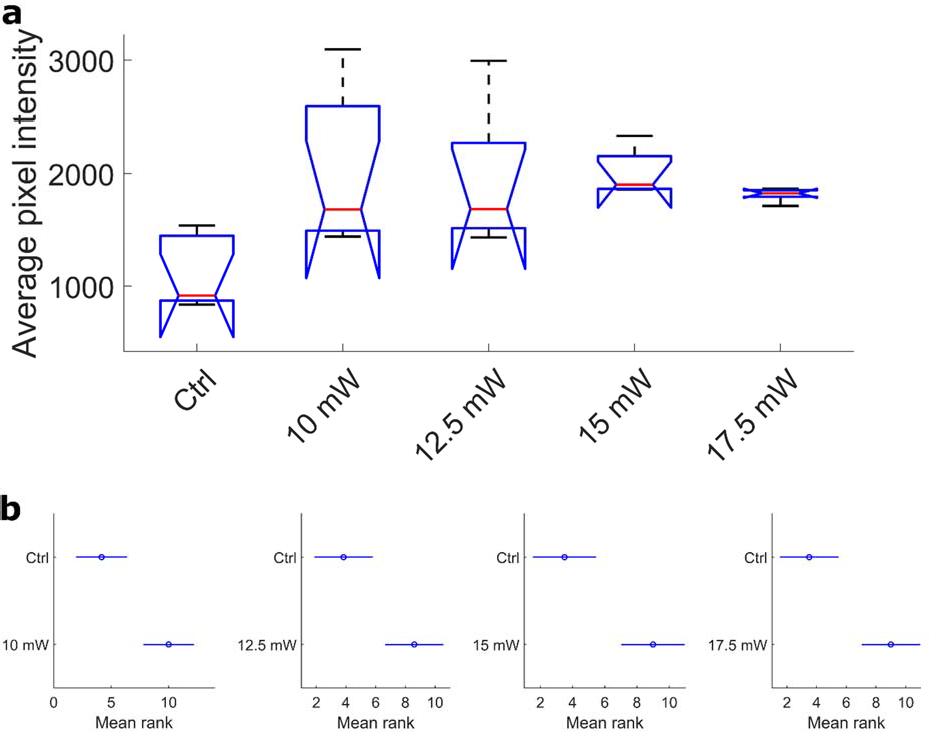


### Fig. S15: Statistical significance of the immunohystochemical analysis in the insertion region.

The average IBA1 intensity in the insertion region is significantly (p<0.05) higher than control in all sample populations from animals that underwent acute implantation. This has been verified by a Kruskal-Wallis test **(a)**, and pairwise Tukey honest significance tests have been performed for protection against type I errors **(b)**.


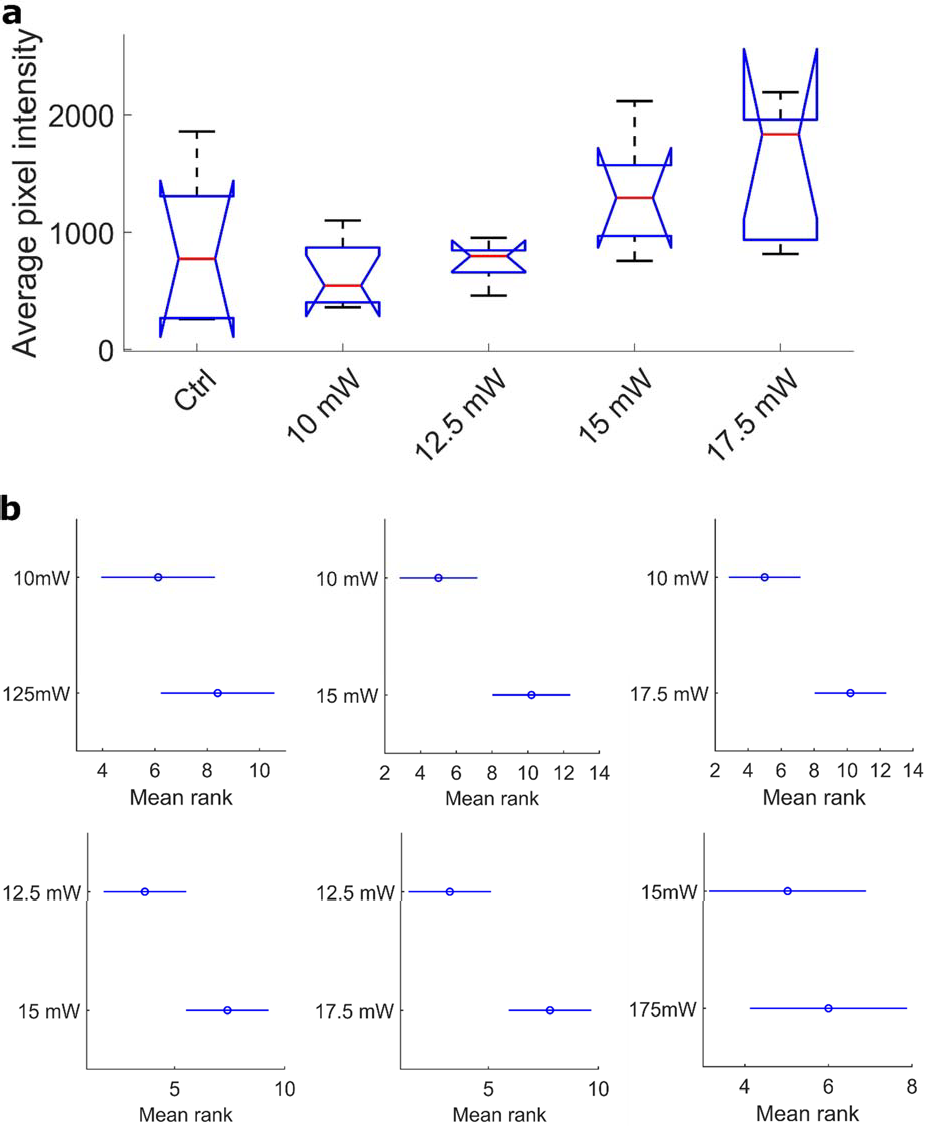


### Fig. S16: Statistical significance of the immunohystochemical analysis in the stimulation region.

The average IBA1 intensity in the stimulation region is not significantly higher than control for any of the sample populations from animals that underwent acute stimulation, while there is a significant difference (p<0.05) between low power (10 and 12.5 mW) and high power (15 and 17.5 mW) stimulations. This has been verified by a Kruskal-Wallis test (**a**), and pairwise Tukey honest significance tests have been performed for protection against type I errors regarding the difference between low and high power (**b**).

### Supplementary Video S2.

Electrophysiology experiment for recording hippocampal theta rhythm in a freely behaving rat. The animal has been chronically implanted with one of the neural interfaces described in this work (left hemisphere) and a steel cannula with tungsten µwires (right hemisphere)


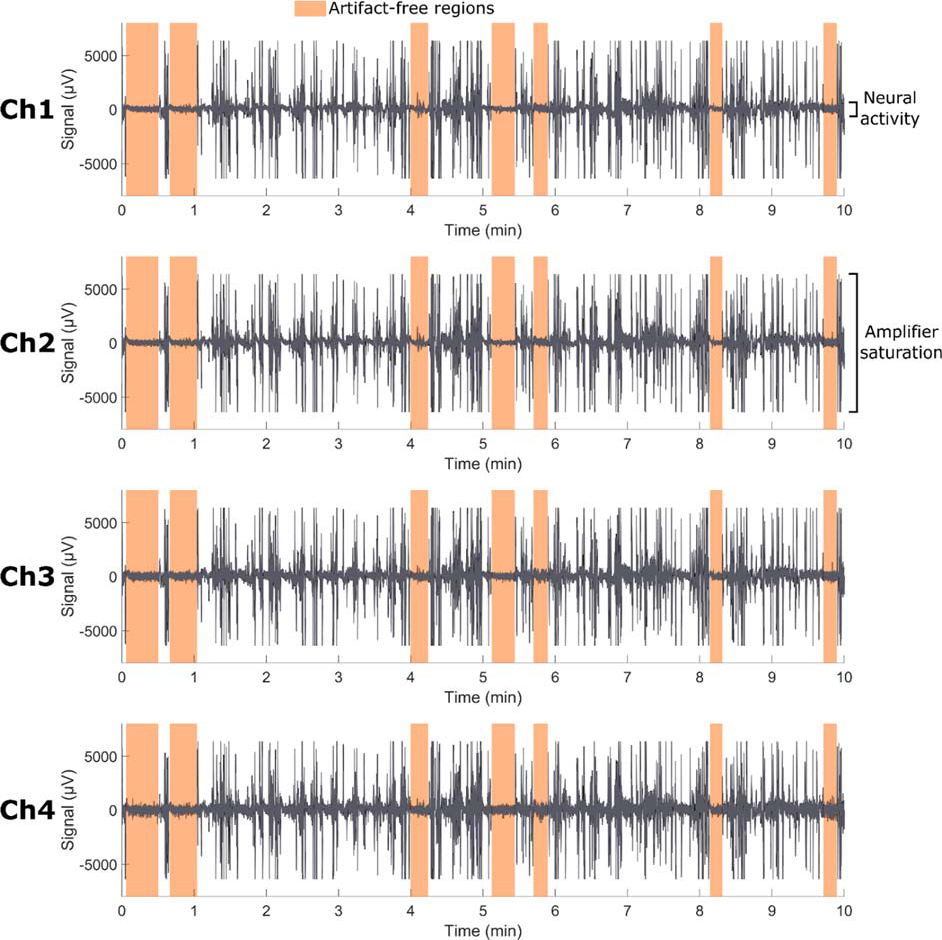


### Fig. S17: Electrophysiological recordings in freely behaving animals.

During electrophysiological recordings in freely behaving rats, we often observed artifacts (attributed to the animal’s motion) with amplitudes orders of magnitude higher than neural activity. It was however possible to extract neural signal from artifact-free regions, as visible from these 10 minutes recordings of hippocampal neural activity in a freely behaving rat after 3 weeks of implantation of a sPOF-based neural interface (channels 1 and 2) and of a cannula with tungsten µwires (channels 3 and 4).


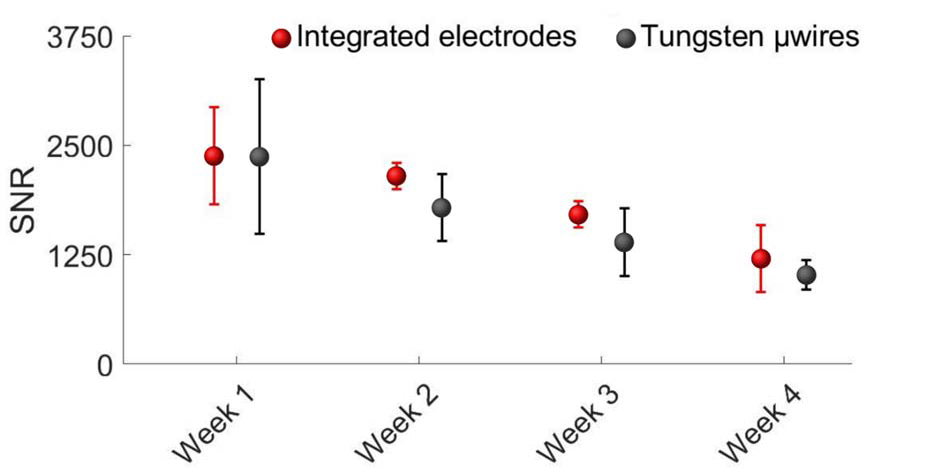


### Fig. S18: Summary of the signal-to-noise ratio comparison between the developed device and standard tungsten µwires in chronic electrophysiology recordings.

The signal-to-noise ratio for the electrophysiological recordings of theta rhythm, while decreasing over time, was comparable between the integrated electrodes and tungsten µwires during the 4 weeks of recording, as visible from the average SNRs and standard errors presented here (n=4 for both electrode types).


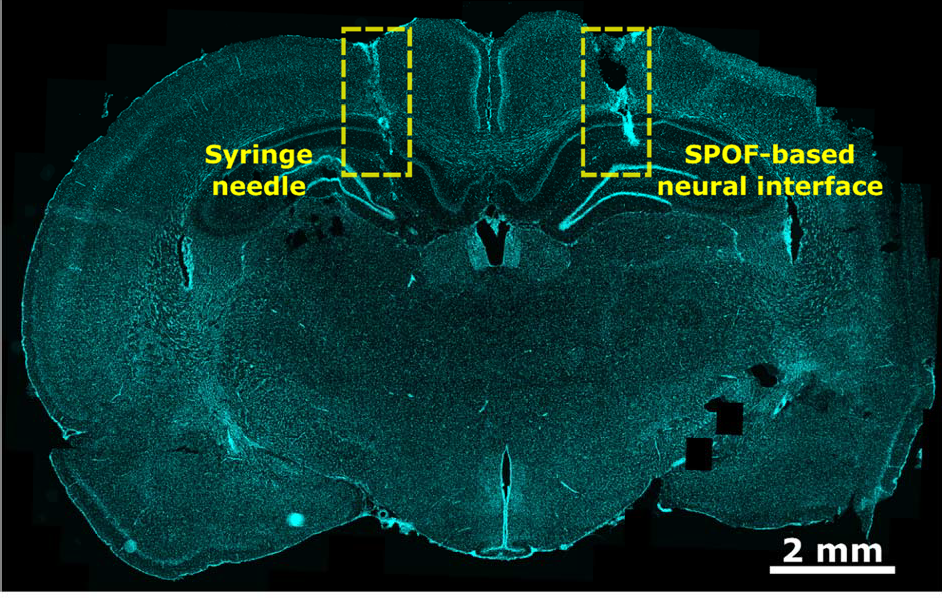


### Fig. S19: Scarring in brain tissue after four weeks of implantation.

The scars left by the two types of chronic implants used in this work were visually evaluated by microscope imaging of brain slices stained with 4′,6-diamidino-2-phenylindole (DAPI). The larger scarring caused by the sPOF-based neural interface is attributed to the difference in shape between the two implants: a flat cleave for the fibre and a sharp edge for the syringe needle.


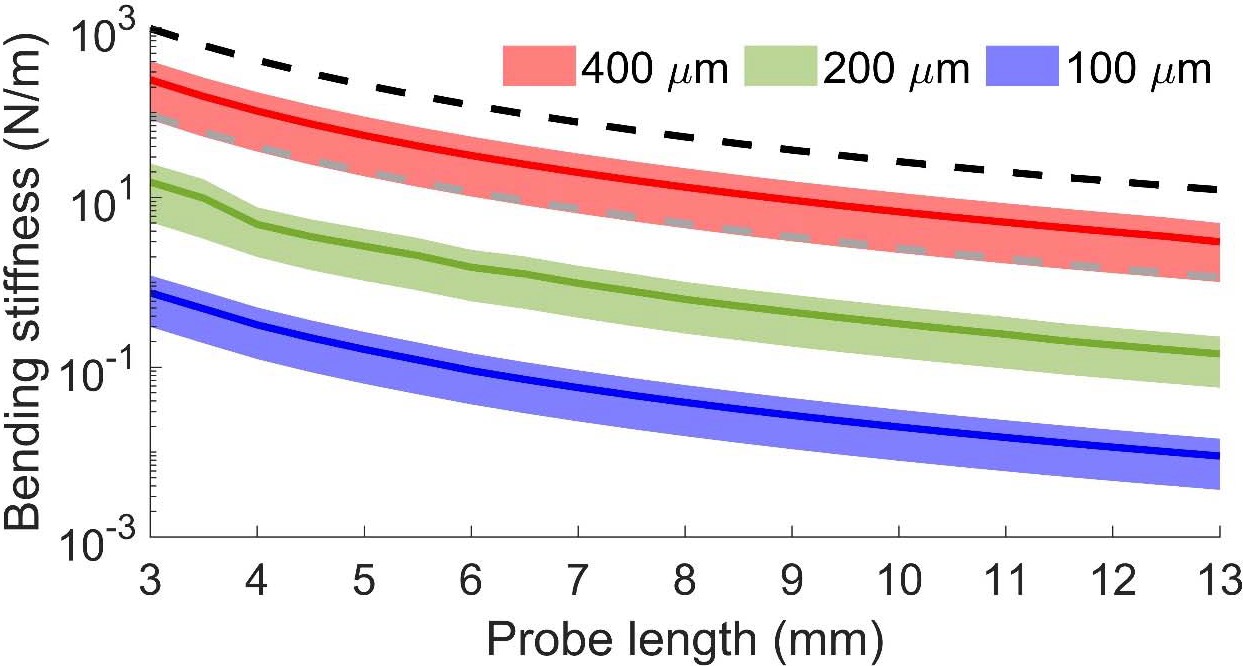


### Fig. S20: Simulated bending stiffness of the indium-filled SPOF with different diameters.

The bending stiffness of the fabricated neural interface can be further reduced by orders of magnitude scaling the fibre down during thermal drawing. As in Figure 1, the calculated values for different diameters are represented as shaded areas due to their geometrical variance, with the average represented as a solid line. The calculated stiffness of silica fibres with diameters of 125 and 225 µm are represented by the gray and black dashed lines, respectively.


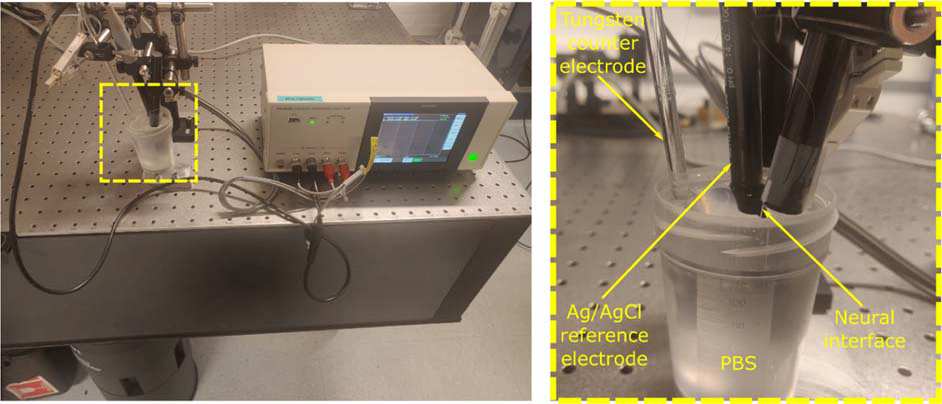


### Fig. S21: Electrochemical impedance spectroscopy setup.

All the impedance spectra reported in this work were acquired using a Hioki IM 3590 chemical impedance analyzer in 3- terminal configuration. In the magnified inset, the location of the neural interface in PBS with respect to the reference and counter electrodes.

# References

21. Antonini, M.J., Sahasrabudhe, A., Tabet, A., Schwalm, M., Rosenfeld, D., Garwood, I., Park, J., Loke, G., Khudiyev, T., Kanik, M., Corbin, N., Canales, A., Jasanoff, A., Fink, Y. & Anikeeva, P. Customizing MRI*‐*Compatible Multifunctional Neural Interfaces through Fiber Drawing. *Advanced Functional Materials* 31, 2104857 (2021).

33. Dickinson, B. L., UDEL® Polysulfone for medical applications. *Journal of Biomaterials Applications* 3, 605-634 (1988).

36. Sui, K., Meneghetti, M., Kaur, J., Sørensen, J .F., Berg, R. W. & Markos, C. Adaptive polymer fiber neural device for drug delivery and enlarged illumination angle for neuromodulation. *Journal of Neural Engineering* 19, 016035 (2022).

58. Wieliczka, D. M., Weng, S. & Querry, M. R. Wedge shaped cell for highly absorbent liquids: infrared optical constants of water. *Applied Optics* 28, 1714-1719 (1989).

59. Sultanova, N. G., Kasarova, S. N. & Nikolov, I. D. Characterization of optical properties of optical polymers. *Optical and Quantum Electronics* 45, 221-232 (2013).

60. Wypych, G., 2022. *Handbook of polymers*. Elsevier.

61. Musgraves, J.D., Hu, J. and Calvez, L. eds., 2019. *Springer handbook of glass* (No. Part A, p. 326). Berlin: Springer.

62. Hull, R. ed., 1999. *Properties of crystalline silicon* (No. 20). IET.

63. Boyer, H.E. and Gall, T.L., 1985. *Metals handbook; desk edition*.

64. Paltauf, G. & Dyer, P. E. Photomechanical processes and effects in ablation. *Chemical reviews* 103, 487-518 (2003).

65. Ma, W., Liu, W. & Li, M. Analytical heat transfer model for targeted brain hypothermia. *International Journal of Thermal Sciences* 100, 66-74 (2016).

66. Azhari, H. *Basics of biomedical ultrasound for engineers* App. A (John Wiley & Sons, New Jersey, 2010)

67. Thompson, A. C., Wade, S. A., Brown, W. G. & Stoddart, P. R. Modeling of light absorption in tissue during infrared neural stimulation. *Journal of biomedical optics* 17, 075002 (2012).
